# RtNAC055 promotes drought tolerance via a stomatal closure pathway linked to methyl jasmonate/hydrogen peroxide signaling in *Reaumuria trigyna*

**DOI:** 10.1093/hr/uhae001

**Published:** 2024-01-03

**Authors:** Binjie Ma, Jie Zhang, Shuyu Guo, Xinlei Xie, Lang Yan, Huijing Chen, Hongyi Zhang, Xiangqi Bu, Linlin Zheng, Yingchun Wang

**Affiliations:** Key Laboratory of Herbage and Endemic Crop Biology, and College of Life Sciences, Inner Mongolia University, Hohhot 010070, China; Institute of Crop Sciences (ICS), Chinese Academy of Agricultural Sciences (CAAS), Beijing 100081, China; Hainan Yazhou Bay Seed Laboratory/National Nanfan Research Institute (Sanya), Chinese Academy of Agricultural Sciences, Sanya 572024, Hainan Province, China; Key Laboratory of Herbage and Endemic Crop Biology, and College of Life Sciences, Inner Mongolia University, Hohhot 010070, China; Key Laboratory of Herbage and Endemic Crop Biology, and College of Life Sciences, Inner Mongolia University, Hohhot 010070, China; Key Laboratory of Herbage and Endemic Crop Biology, and College of Life Sciences, Inner Mongolia University, Hohhot 010070, China; Institute of Crop Sciences (ICS), Chinese Academy of Agricultural Sciences (CAAS), Beijing 100081, China; Hainan Yazhou Bay Seed Laboratory/National Nanfan Research Institute (Sanya), Chinese Academy of Agricultural Sciences, Sanya 572024, Hainan Province, China; Institute of Crop Sciences (ICS), Chinese Academy of Agricultural Sciences (CAAS), Beijing 100081, China; Hainan Yazhou Bay Seed Laboratory/National Nanfan Research Institute (Sanya), Chinese Academy of Agricultural Sciences, Sanya 572024, Hainan Province, China; Key Laboratory of Herbage and Endemic Crop Biology, and College of Life Sciences, Inner Mongolia University, Hohhot 010070, China; Key Laboratory of Herbage and Endemic Crop Biology, and College of Life Sciences, Inner Mongolia University, Hohhot 010070, China; Key Laboratory of Herbage and Endemic Crop Biology, and College of Life Sciences, Inner Mongolia University, Hohhot 010070, China; Key Laboratory of Herbage and Endemic Crop Biology, and College of Life Sciences, Inner Mongolia University, Hohhot 010070, China

## Abstract

The stomata regulate CO_2_ uptake and efficient water usage, thereby promoting drought stress tolerance. NAC proteins (NAM, ATAF1/2, and CUC2) participate in plant reactions following drought stress, but the molecular mechanisms underlying NAC-mediated regulation of stomatal movement are unclear. In this study, a novel NAC gene from *Reaumuria trigyna*, *RtNAC055*, was found to enhance drought tolerance via a stomatal closure pathway. It was regulated by RtMYC2 and integrated with jasmonic acid signaling and was predominantly expressed in stomata and root. The suppression of *RtNAC055* could improve jasmonic acid and H_2_O_2_ production and increase the drought tolerance of transgenic *R. trigyna* callus. Ectopic expression of *RtNAC055* in the *Arabidopsis atnac055* mutant rescued its drought-sensitive phenotype by decreasing stomatal aperture. Under drought stress, overexpression of *RtNAC055* in poplar promoted ROS (H_2_O_2_) accumulation in stomata, which accelerated stomatal closure and maintained a high photosynthetic rate. Drought upregulated the expression of *PtRbohD/F*, *PtP5CS2*, and *PtDREB1*.*1*, as well as antioxidant enzyme activities in heterologous expression poplars. RtNAC055 promoted H_2_O_2_ production in guard cells by directly binding to the promoter of *RtRbohE*, thus regulating stomatal closure. The stress-related genes *RtDREB1*.*1/P5CS1* were directly regulated by RtNAC055. These results indicate that RtNAC055 regulates stomatal closure by maintaining the balance between the antioxidant system and H_2_O_2_ level, reducing the transpiration rate and water loss, and improving photosynthetic efficiency and drought resistance.

## Introduction

Plant growth and yield are severely affected by drought. The effect of drought on crop and woody plant biomass is increasing worldwide, especially in arid and semiarid regions, as a result of climate change problems, such as limited rainfall, excessive vaporization, and global warming [1, 2]. The adverse effects of drought are mediated by membrane damage, osmotic stress, and reduced photosynthetic and respiratory rates, which cause retardation of plant growth and metabolism [3]. In the leaf epidermis, guard cells are arranged in pairs, forming stomatal pores that mediate entry of CO_2_ (photosynthetic raw material) together with water transpiration [4]. Stomatal transpiration contributes to ~95% of overall plant-based water losses [5]. Stomatal closure reduces water loss under drought conditions. Movement of guard cells is induced by reactive oxygen species (ROS), abscisic acid (ABA), methyl jasmonate (MeJA), CO_2_ and water status, and controls both transpiration and photosynthesis [6–8].

ROS, involved in stomatal closure regulation, comprise ubiquitous metabolites produced in all organisms [9]. Their accumulation in the apoplast and chloroplast serves as the earliest signs of stomatal closure. Excess ROS first accumulate in the guard cell apoplast, and the subsequent sensing and signaling activities contribute to the activation of K^+^/Ca^2+^ channels [10]. As reported for ABA, MeJA can induce accumulation of ROS in *Arabidopsis thaliana* guard cells, and this process is regulated by plasma membrane NAD(P)H oxidase Atrboh/F. Next, ROS act as signaling molecules in the plasma membrane and activate non-selective Ca^2+^-permeable cation channels [11–15]. MYC2, a bHLH transcription factor (TF), the master TF in JA signaling, is essential in a variety of JA-induced processes, such as lateral root development [16], saline stress [17], cold stress response [18], wounding/pathogen invasion [19, 20], leaf and fruit senescence [21–24], plant photomorphogenesis [25], ROS accumulation [26], and secondary metabolite biosynthesis [23, 27–29]. MYC2s participate in the mediation of responses against drought stress [30, 31], though such a regulatory network and direct target gene(s) involved in the response to stomatal opening and closure under drought stress are unclear.

Environmental stress perception and transmission lead to the activation of transcriptional control [32]. NAC, MYB/MYC, WRKY, DREB, and nuclear factor TFs are implicated in reactions following abiotic/biotic stress conditions [33–43]. The TFs involved in guard cell movements in plants have been reported [44–48]. The JAZ2 mutations in *Arabidopsis* restrain stomatal reopening by coronatine (COR) and promote resistance to bacterial invasion. In addition, MYC2/3/4 as JAZ2 target genes directly control *ANAC19*/55/72 expression and modulate the stomatal aperture [49]. Two homologous NAC TFs in tomato, JA2 and JA2-like (JA2L), are specifically expressed in guard cells. JA2 regulates ABA accumulation and is implicated in ABA-meditated guard cell movement, and JA2L inhibits salicylic acid biosynthesis by regulating SA metabolism-related genes and controls pathogen-triggered stomatal closure [50]. Loss of function of MYB61, an *Arabidopsis* R2R3-MYB TF predominantly expressed in guard cells, reduces stomatal aperture, together with reductions in water loss and wilting symptoms during drought stress [51]. Repression of DST, a C2H2 zinc finger TF, results in downregulation of H_2_O_2_ homeostasis-related genes such as peroxidase 24 precursor, whose promoter contains a DST-binding element. This event triggers H_2_O_2_ production and accelerates stomatal closure, consequently promoting drought/salt tolerance within *Oryza sativa* [52]. In many species, NAC TFs are implicated in the regulation of guard cell movement. For instance, the stress-response NAC1, *SNAC1*, controls the expression of the SRO protein OsSRO1c specifically produced in stomata under abiotic stress. Overexpression of *OsSRO1c* lowers transpiration-mediated water loss via triggering H_2_O_2_ production in guard cells, which reduces the aperture of open stomata [53]. Under drought stress, *MusaSNAC1* expression is upregulated in guard cells of banana, where it triggers stomatal closure by elevating the H_2_O_2_ concentration in the guard cells [54]. However, the network of NAC TFs implicated in stomatal movement in halophytes, particularly recretohalophytes, is unclear.

Inner Mongolia is a harsh environment characterized by high soil salinity (up to 0.7% salts), drought (annual average precipitation 140.9–302.2 mm), low temperatures (annual average temperature 6.0–9.2°C), and scorching summers (maximum surface temperature 68.5°C) [55–57]. Many plants with tolerance to extreme environments grow in this area, and an in-depth study of the drought tolerance mechanisms of recretohalophytes would accelerate the generation of drought-resilient plants for use in agriculture [42, 58]. To adapt to these environmental stresses, *Reaumuria trigyna* has evolved distinct morphological and physiological features, including succulent, acicular leaves, salt-excreting glands, a highly efficient antioxidant system, and strong osmoregulatory capacity [59–61]. *Reaumuria trigyna* is a typical recretohalophyte in this area and can act as a reference species for those that thrive in the severely adverse environment of the Eastern Alxa–Western Or dos desert. In previous studies, we identified 35 NAC TFs from a transcriptome database of *R*. *trigyna*. RtNAC100 was implicated in mediating salt tolerance by triggering ROS accumulation and programmed cell death (PCD) induction in plants [42]. However, no study has investigated the role of NAC TFs in stomatal movements during drought stress in *R*. *trigyna*. In this study, an NAC TF, *RtNAC055*, was identified from *R*. *trigyna*, and was predominantly expressed in guard cells and directly regulated by MYC2. RtNAC055 regulated the expression of *RtRbohE/DREB1*.*1/P5CS1* by directly binding to their promoters, which increased H_2_O_2_ accumulation in stomata, maintained the balance between the antioxidant system and ROS level, and promoted stomatal closure. Thus, plant drought tolerance is enhanced by a reduction in the transpiration rate and water loss, maintenance of the balance between the antioxidant system and ROS level, and promotion of photosynthetic efficiency.

## Results

### Jasmonic acid biosynthesis induced by drought and expression patterns of *RtNAC055* in *R*. *trigyna*

JA biosynthesis-related genes were upregulated in *R*. *trigyna* seedlings after 400 mM NaCl treatment [42]. In order to probe JA activity following *R*. *trigyna* drought stress, qRT–PCR was performed. PEG treatment led to a significant upregulation in JA biosynthesis-related gene expression (*RtMYC2/AOS1*.*2/AOC4/LOX3/AOS1*.*1*) in leaves (Supplementary Data Fig. S1), implicating MeJA in the drought response of *R*. *trigyna*.

**Figure 1:**
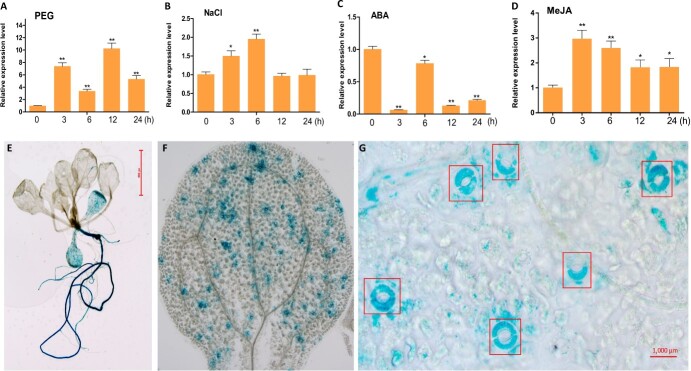
*RtNAC055* expression pattern and GUS staining of *proRtNAC055* transgenic *Arabidopsis* seedlings. **A**–**D***RtNAC055* expression under PEG, NaCl, ABA, and MeJA treatment, respectively. Samples were from *R*. *trigyna* seedlings. ^*^P < 0.05, ^**^P < 0.01; Student’s *t*-test. **E**–**G** GUS staining of transgenic *Arabidopsis* seedlings (2 weeks old), leaf, and stomata (red boxes).

The role of AtNAC055 in MeJA-related biological functions has been investigated [27, 49]. Bioinformatics analysis showed that RtNAC055 had a conserved NAM domain at amino acids 1–150 and had higher homology with AtNAC055 than other NAC proteins in *Arabidopsis* (Supplementary Data Figs S2 and S3). In this work, *RtNAC055* expression was upregulated in increasing order by PEG, NaCl, and exogenous MeJA (Fig. 1A). *RtNAC055* expression first increased and decreased thereafter following NaCl and PEG exposure, with peaks at 12 and 6 h. Exogenous MeJA induced* RtNAC055* expression, which peaked at 3 h (Fig. 1D). Therefore, *RtNAC055* was induced by JA and environmental stresses, and may be an upstream signal in the regulation of *RtNAC055* expression following drought/salinity stress.

Analysis for *RtNAC055* promoter recognized multiple putatively stress-response *cis*-elements, including LTRs, ABREs, drought-related MYB-binding sites, TATC boxes, GARE motifs, and TGACG motifs (Supplementary Data Table S1). We determined the tissue localization of gene expression by transferring the *RtNAC055* promoter into Columbia-0. A GUS staining assay showed that RtNAC055 was predominantly expressed in roots and stomatal guard cells, implicating this in root development and stomatal movement (Fig. 1E–G).

### RtNAC055 serves as a transcriptional activator

To evaluate nuclear RtNAC055 localization, recombinant vectors 35S::GFP and 35S::RtNAC055-GFP were transiently expressed in *Nicotiana benthamiana* leaf epidermal cells. DAPI staining revealed 35S::RtNAC055-GFP fluorescence in nuclei. However, 35S::GFP control signal was distributed throughout the cell (Supplementary Data Fig. S4A). Hence, RtNAC055 encodes a nuclear protein.

Yeast AH109 harboring GALBD-RtNAC055 vector grew on SD/−Trp and SD/−Trp/−His/−Ade medium, demonstrating positive galactosidase function. Conversely, the negative control (CK−) transformants expressing pGBKT7 empty vectors failed to grow (Supplementary Data Fig. S4B). Therefore, RtNAC055 has transactivation activity in yeast cells.

NAC TFs modulate target gene expression by bonding to NACRS (CGT[G/A]) *cis*-elements in promoter regions. A yeast one-hybrid (Y1H) assay showed that transformants harboring pGADT7-RtNAC055 and pAbAi-NACRS, but not the negative control, thrived on SD/−Ura/−Leu medium plus AbA (Supplementary Data Fig. S5). Therefore, RtNAC055 binds to CGT[G/A] *cis*-elements to transactivate reporter genes in yeast.

### RtNAC055 improves drought tolerance of transgenic *R. trigyna* callus and *Arabidopsis*

**Figure 2:**
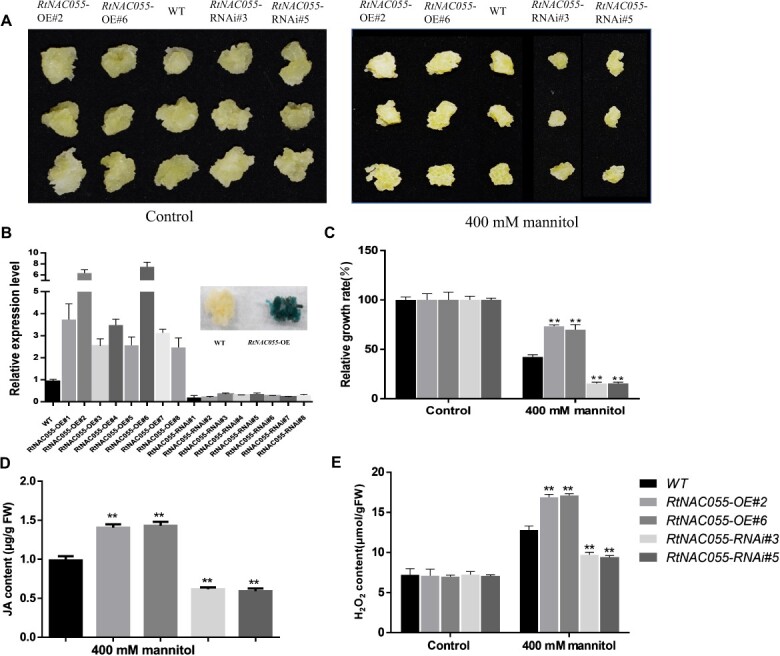
*RtNAC055* promoted JA and H_2_O_2_ synthesis and drought tolerance of transgenic *R. trigyna* callus. **A** Morphological characteristics of WT, *RtNAC055* OE, and RNAi, transgenic callus under mannitol treatment. **B** Relative expression level of *RtNAC055* in WT and transgenic callus. **C** Relative growth rate of *R. trigyna* callus under mannitol treatment. **D**, **E** JA and H_2_O_2_ contents of transgenic *R. trigyna* callus under mannitol treatment. ^*^P < 0.05,^**^P < 0.01; Student’s *t*-test.

To investigate the function of RtNAC055, we obtained transgenic *R. trigyna* callus of *RtNAC055.* We carried out real-time fluorescence quantification and a GUS staining assay to certify that *RtNAC055*-overexpressing (OE) and RNAi vector were transferred into callus of *R. trigyna* successfully (Fig. 2B). Then we analyzed the tolerance of different lines of *R. trigyna* callus to mannitol treatments. Wild-type (WT) and *RtNAC055* OE/RNAi callus of the same size and fresh weight were grown on MS medium without or containing 400 mM mannitol for 15 days, and then the callus phenotypes and growth rates were analyzed. The growth rates of the WT and *RtNAC055* OE/RNAi grape callus on MS medium were similar (Fig. 2A). Compared with the WT, the *RtNAC055* RNAi transgenic callus was more sensitive to mannitol, and the growth rate was slower. However, the *RtNAC055* OE callus had higher tolerance of mannitol and a faster growth rate (Fig. 2C). JA and H_2_O_2_ contents in OE *RtNAC055* callus were higher under mannitol treatment than in the WT and *RtNAC055* RNAi groups (Fig. 2D and E). Therefore, our results suggested that RtNAC055 accelerated JA and H_2_O_2_ synthesis and enhanced tolerance of mannitol in *R. trigyna*.

**Figure 3:**
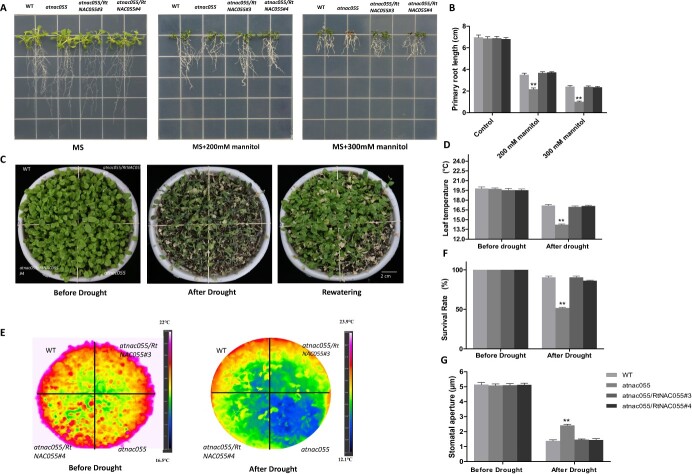
**A** Root length of *RtNAC055* transgenic *Arabidopsis* seedlings under mannitol treatment and phenotype under drought. **B** Primary root length under drought. **C**, **D** Phenotype and survival rate of *RtNAC055* transgenic plants under drought or rewatering conditions. **E**, **F** Leaf surface temperature before and after drought. **G** Stomatal aperture. ^*^P < 0.05, ^**^P < 0.01; Student’s *t*-test.

AtNAC055 is implicated in drought tolerance [70, 71]. To investigate RtNAC055 function, a vector containing *RtNAC055* was introduced into the *atnac055* mutant to generate complementary lines. After drought treatment, Col-0 and the complementary lines showed longer roots and enhanced stress tolerance compared with *atnac055* plants, and the drought-sensitive phenotype of mutant lines was rescued in the complementary lines (Fig. 3A and B).

Because water loss by transpiration decreases the temperature of the leaf surface, we measured temperature in Col-0, *atnac055* mutant, and *atnac055-*OE *RtNAC055* plants. The leaf temperature and survival rate of the *atnac055* mutant were lower than those of Col-0 and *atnac055-*OE *RtNAC055* plants under drought treatment. Overexpression of *RtNAC055* rescued the phenotype of leaf surface temperature reduction in the mutant (Fig. 3C–F).

Stomatal closure was insensitive to drought in the *atnac055* mutant. To determine whether *RtNAC055* regulates stomatal closure in the transgenic lines under drought treatment, we examined the stomatal aperture in *atnac055*-OE RtNAC055 plants. The insensitive phenotype of stomatal closure of *atnac055* in response to drought was partially rescued (Fig. 3G and Supplementary Data Fig. S6).

The above results indicate that RtNAC055 restored the temperature of the leaf surface of complementary lines by reducing the stomatal aperture and decreasing transpiration and water loss. This increased the survival rate of *atnac055*-OE *RtNAC055* plants under drought treatment (Fig. 3F).

### *RtNAC055* overexpression improves water use efficiency and photosynthetic rate by reducing stomatal conductance

**Figure 4:**
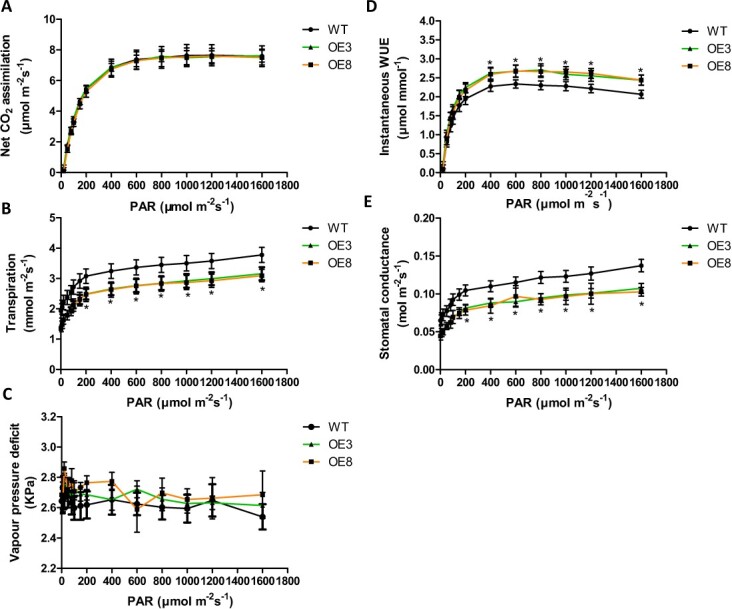
Light-response curves of *RtNAC055* transgenic and WT poplar under normal conditions. **A** Net CO_2_ assimilation. **B** Transpiration rate. **C** Vapor pressure deficit. **D** instantaneous WUE. **E** Stomatal conductance. ^*^P < 0.05, ^**^P < 0.01; Student’s *t*-test.

Stomatal movement is closely related to water use efficiency (WUE) and photosynthesis. To clarify the function of RtNAC055 in pant drought tolerance, a recombinant plasmid harboring the *RtNAC055* coding sequence drove through the 35S promoter was introduced into poplar. GUS stain-step, genomic PCR, and qRT–PCR were performed to identify *RtNAC055* transgenic poplar (Supplementary Data Fig. S8). To determine whether RtNAC055 regulates photosynthesis, we generated photosynthesis–light curves for WT and OE *RtNAC055* poplar under normal watering conditions. The net CO_2_ assimilation was similar for OE lines and WT (Fig. 4A). Leaf transpiration rate and stomatal conductance were markedly lower for OE lines in comparison with WT (Fig. 4B and E). Furthermore, OE lines demonstrated higher instantaneous WUE than the WT (Fig. 4D). No major variations were found within vapor pressure deficit across WT and OE lines (Fig. 4C), indicating that transpiration rate was unaffected by vapor pressure deficit. We speculate that RtNAC055 regulates stomatal movements to reduce the transpiration rate and increase the WUE and photosynthetic rate.

### RtNAC055 contributes positively to drought tolerance of transgenic poplars

**Figure 5:**
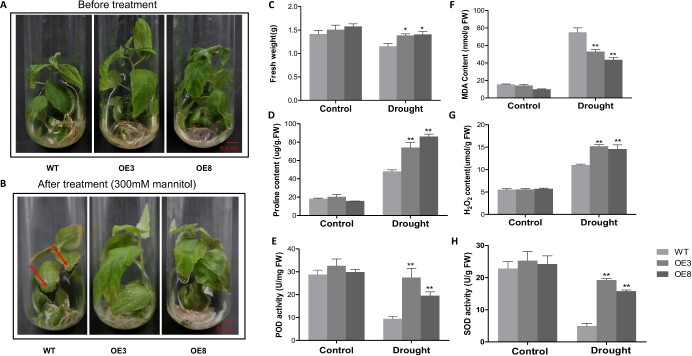
Drought tolerance of *RtNAC055* transgenic plants. **A**, **B** Poplar phenotype under normal conditions and 300 mM mannitol treatment. **C** Fresh weight. **D** Proline content. **E** POD activity. **F** MDA level. **G** H_2_O_2_ level. **H** SOD activity. ^*^P < 0.05, ^**^P < 0.01; Student’s *t*-test. Scale bar, 0.5 cm.

OE and WT plantlets were cultured in liquid Hoagland media supplemented with 300 mM mannitol. After 10 days some leaves from the WT group had died and fresh weight had decreased. By contrast, OE3 and OE8 plants exhibited better growth with little discoloration (Fig. 5A and B). In addition, the proline and H_2_O_2_ contents and POD/SOD activities were elevated for OE lines in comparison with WT after drought treatment; by contrast, the MDA content was lower (Fig. 5C–H). Therefore, *RtNAC055* overexpression enhanced the drought tolerance of poplar.

**Figure 6:**
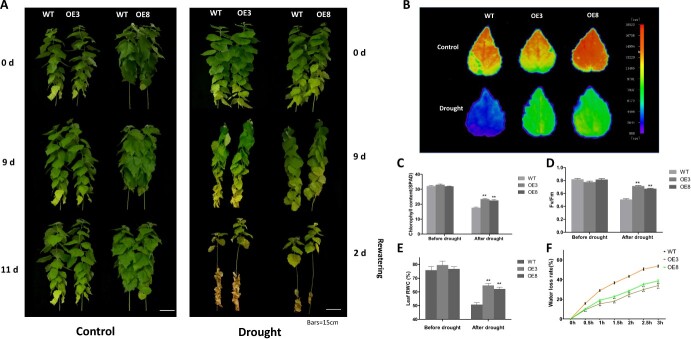
Physiological and biochemical parameters of WT and transgenic poplar under drought stress. **A** Poplar phenotype following 9 days of drought and 2 days of rewatering. **B** DF images. **C** Chlorophyll content. **D***F*_v_/*F*_m_ value. **E** Leaf RWC. **F** Water loss rate. ^*^P < 0.05, ^**^P < 0.01; Student’s *t*-test. Scale bar, 15 cm.

To further analyze RtNAC055 function under drought stress, a short-term drought stress in soil assay was performed. On day 9, WT plant leaves were seriously wilted and had a decreased chlorophyll content, whereas OE poplar leaves remained turgid. After rewatering for 2 days, OE plant leaves seemed refreshed and remained upright, whereas WT plant leaves were severely wilted, with abscission and senescence (Fig. 6A). Moreover, the leaf relative water content (RWC) for OE plants was elevated in comparison with WT plants after drought stress (Fig. 6E).

After 9 days of drought stress, *F*_v_/*F*_m_, leaf RWC, and chlorophyll content were significantly higher while water losses were reduced for OE lines in comparison with WT (Fig. 6). By contrast, there was little difference in these parameters under normal conditions. Delayed fluorescence (DF) is predominantly emitted from PSII, which is a sensitive phenomenon highly associated with changes in different photosynthetic processes induced by environmental factors. After drought treatment, DF signals for OE poplar leaves were stronger than those for the WT (Fig. 6B), indicating that PS II had a higher chlorophyll content and photosynthetic efficiency. Therefore, RtNAC055 enhanced the drought tolerance of transgenic poplar.

### RtNAC055 promotes H_2_O_2_-induced stomatal closure

**Figure 7:**
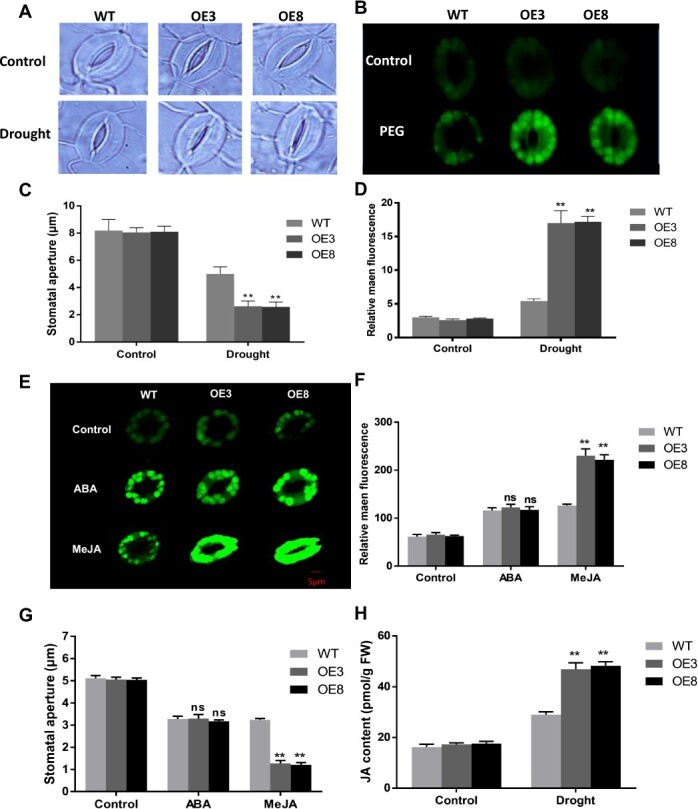
MeJA and drought induced stomatal closure by triggering production of ROS in *RtNAC055 *transgenic poplar. **A**, **C** Stomatal closure of WT and transgenic poplar under drought. **B**, **D** ROS accumulation and fluorescence quantification in WT and transgenic poplar under PEG treatment. **E**, **F** Representative images and fluorescence quantification of ROS in guard cells. **G** Stomatal aperture in WT and transgenic poplar after 30 min of ABA and MeJA treatment. **H** JA content in WT and transgenic poplar after 30 min of PEG treatment. ^*^P<0.05,^**^P < 0.01; ns, no significant difference; Student’s *t*-test. Scale bar, 5 μm.

H_2_O_2_ is a valuable secondary messenger implicated in stomatal closure under abiotic stresses [9]. To evaluate the role of RtNAC055 in H_2_O_2_ production to induce stomatal closure, we used 2,7-dichlorodihydrofluorescein diacetate (H_2_DCFDA) to measure the endogenous H_2_O_2_ level in stomatal guard cells of WT and OE poplars under PEG treatment. Under normal conditions, H_2_O_2_ content was similar for both plant types. Following PEG introduction the levels of H_2_O_2_ increased in WT and OE lines. OE lines had a considerably higher H_2_O_2_ level than WT plants in stomatal guard cells and leaves (Fig. 7B). Stomatal aperture was smaller in transgenic poplar in comparison with WT during drought stress (Fig. 7A). Therefore, RtNAC055 accelerated H_2_O_2_ production in stomata, triggering stomatal closure.

**Figure 8:**
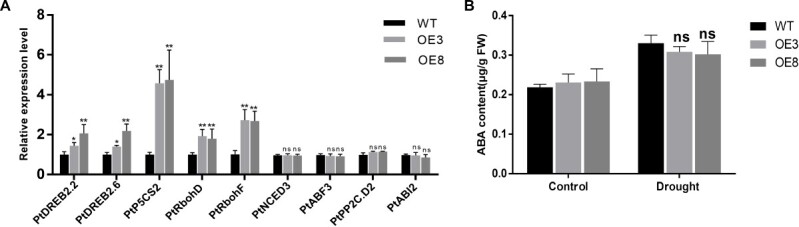
Stress-response gene expression levels and ABA contents in WT and OE *RtNAC055* poplar under drought stress. Poplars were grown for 4 weeks in tissue-culture vessels and treated with 300 mM mannitol for 5 days. *PtACTIN1* was the internal control. ^*^P < 0.05; ^**^P < 0.01; ns, no significant difference; Student’s *t*-test,

We investigated time-course changes in the signaling events under ABA or JA treatment. Within 30 min after stimulation with ABA, the ROS production of stomata in transgenic and WT poplar was significantly induced, but there were no remarkable differences between different lines (Fig. 7E and F). However, MeJA could induce more ROS production of stomata in transgenic lines than WT poplar. Thus, MeJA content increased within 30 min in transgenic poplar after drought treatment to induce ROS production and stomatal closure (Fig. 7G and H). ABA content in OE *RtNAC055* poplar was not significantly different between the PEG treatment group and the WT group (Fig. 8B). Meanwhile, we investigated the gene expression level in the ABA signaling pathway (*PtNCED3/ABF3/ABI2/PP2C.D2*) under drought treatment. The expression of ABA biosynthesis-related genes in leaves was not significantly induced in transgenic poplar after drought treatment (Fig. 8A). Therefore, we speculated that ABA may not be involved in the signaling to regulate *RtNAC055* and enhance drought tolerance. The DREB, P5CS, and RBOH genes are drought-responsive and promote drought tolerance. The expression levels of *PtP5CS2*, *PtDREB2*.*2/2*.*6*, and *PtRbohD/F* in OE poplars were upregulated significantly by drought stress.

### RtNAC055 interacts directly with *RtRbohE/DREB1*.*1/P5CS1* and RtMYC2 affects *RtNAC055* expression by directly binding to the promoter

NAC TFs activate the drought response by regulating stomatal closure, modulating antioxidant activities, and promoting proline accumulation [71–74]. Bioinformatic analysis revealed a potential NAC-binding motif (CGT[A/G]) in *RtRbohE*, *DREB1*.*1*, and *P5CS1* promoters. qRT–PCR showed that *RtRbohE*, *DREB1*.*1*, and *P5CS1* expression was significantly induced by drought (Supplementary Data Fig. S7).

**Figure 9:**
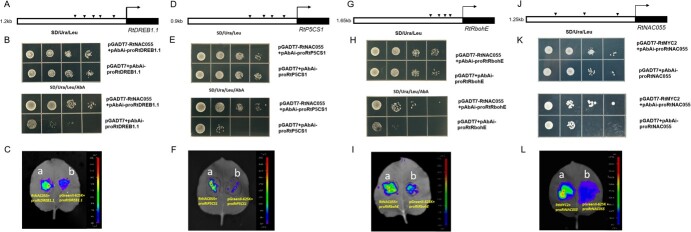
Yeast one hybrid and transient expression assays showing that RtNAC055 promoted the expression of *RtDREB1*.*1*, *RtP5CS1*, and *RtRbohE*. **A**, **D**, **G**, **J** NACRS analysis of target gene promoters. **B**, **E**, **H**, **K** Y1H assay. **C**, **F**, **I**, Dual-luciferase assay. a, pro*RtDREB1*.*1/P5CS1/RbohE*-LUC + RtNAC055-pGreenII-62 SK; b, pro*RtDREB1*.*1/P5CS1/RbohE*-LUC + pGreenII-62SK; **L**: a, (pro*RtNAC055*-LUC *+* RtMYC2-pGreenII-62SK); b, (pro*RtNAC055*-LUC *+* pGreen-62IISK).

Y1H assays showed that after co-transformation of RtNAC055 and the promoter of *RtRbohE*, *DREB1*.*1*, and *P5CS1*, Y1H yeast grew normally in selective medium but not control medium. Therefore, RtNAC055 binds to the NACRS promoter in target genes. Dual-luciferase assays showed that coexpression of 35S::RtNAC055 and *RtRbohE*, *DREB1*.*1*, and *P5CS1pro*::LUC significantly increased luminescence intensity, which was decreased by pGreen-62sk empty vector and *RtRbohE*, *DREB1*.*1*, and *P5CS1pro:*:LUC as controls (Fig. 9). Therefore, RtNAC055 positively regulates *RtRbohE*, *DREB1*.*1*, and *P5CS1* expression by directly binding to their promoters.

**Figure 10:**
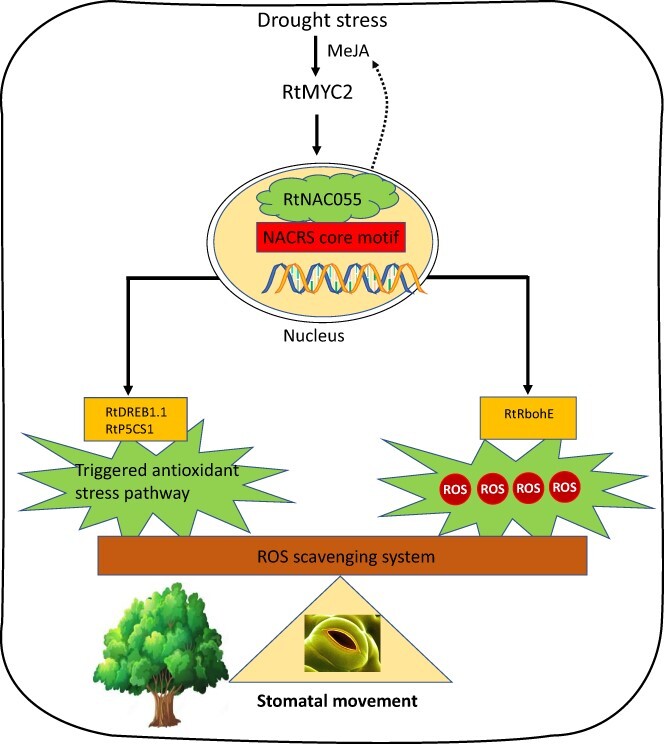
Transcriptional regulatory pathways involved in RtNAC055 under drought stress. Under drought stress the JA level increases and leads to activation of the RtMYC2-mediated JA signaling pathway in plant cells and stimulation of *RtNAC055* transcription, which promotes the expression of the ROS biosynthesis gene *RtRbohBE* and accumulation of ROS in guard cells to mediate the closure of stomata for drought resistance. *RtNAC055* upregulates the expression of the proline synthesis gene *RtP5CS1* and the drought-related gene *RtDREB1*.*1*, triggering proline production and increasing antioxidant activity. RtNAC055 modulates drought resistance by maintaining the oxidant–antioxidant balance in a manner mediated by MeJA.

MYC2 is a bHLH TF, the master TF in JA signaling, typically bound to the G-box (CACGTG) in their target genes [75]. In *Arabidopsis*, MYC2 directly regulates* ANAC019/055/072* expression by binding specifically to the promoter of the MYC recognition sequence (G-box) [27, 49]. Bioinformatic analysis identified three potential MYC recognition elements (G-box) in the promoter region of *RtNAC055*. Y1H and dual-luciferase assays demonstrated RtMYC2 directly regulates *RtNAC055* expression (Fig. 9). Therefore, RtMYC2 may bind to G-box elements to regulate *RtNAC055* expression.

## Discussion

TFs function in senescence [42, 76, 77], root formation [78, 79], fruit ripening [22, 80–82], secondary cell wall development [74, 83–85], and responses to biotic/abiotic stress [37, 38, 86–88]. Some stress-related TFs enhance plant abiotic stress tolerance by directly regulating stomatal movements and modulating photosynthesis. For instance, PdGNC directly regulates *PdHXK1* expression in poplar to mediate stomatal closure through NO and H_2_O_2_ production in guard cells, thereby enhancing WUE and improving drought tolerance [68]. PeABF3 enhances drought tolerance and maintains high photosynthetic activity by directly regulating *PeADF5* expression and triggering ABA-induced stomatal closure [66]. A salicylic acid biosynthesis-related TF, *PtrWRKY75*, stimulates ROS accumulation in leaves and increases the expression of the salicylic acid biosynthesis gene *PtrPAL1*. This phenomenon results in stomatal closure and reduction in transpiration, eventually enhancing WUE and drought tolerance [89]. NAC TFs are implicated in the regulation of stomatal movements and photosynthesis, particularly NAC055 in *Arabidopsis* and *Brassica napus*. In *Arabidopsis*, AtNAC055 promotes the drought response by enhancing proline accumulation to regulate stomatal closure [71]. BnaNAC55 triggers ROS biosynthesis and defense-related genes, inducing ROS accumulation and hypersensitive response-like cell death against biotic stress [90]. In this study, RtNAC055, which has higher homology to AtNAC055 than other NAC proteins in *Arabidopsis*, was isolated from *R*. *trigyna*, a typical recretohalophyte thriving in the Eastern Alxa–Western Ordos Desert. The expression of *RtNAC055* was induced by drought, salt, and MeJA, and its promoter harbored stress-related *cis*-elements. The transgenic *R. trigyna* callus assay suggested that RtNAC055 accelerated JA and H_2_O_2_ synthesis and enhanced tolerance of mannitol in *R. trigyna*. *RtNAC055* was transfected into *atnac055* mutant *Arabidopsis* and *Populus davidiana* × *P*. *bolleana*. The complementary lines had a narrower stomatal aperture and higher leaf surface temperature and survival rate than the *atnac055* mutant under drought, which suggests that RtNAC055 rescued the drought-sensitive phenotype of the *atnac055* mutant via regulation of stomatal movements. Compared with the WT, transgenic poplar overexpressing *RtNAC055* showed higher drought tolerance and had higher proline content and SOD/POD activities and a lower MDA level. In addition, it had higher chlorophyll and RWC contents and lower water loss, increasing photosynthetic efficiency and *F*_v_/*F*_m_, and DF with PSII under drought stress. Drought markedly upregulated a proline synthesis-related gene (*PtP5CS2*), ROS production-related genes (*PtRbohD/F*), and dehydration-responsive genes (*PtDREB2*.*2/2*.*6*) in transgenic poplar. In addition, H_2_O_2_ accumulation in stomatal guard cells was enhanced in the OE lines under drought, accelerating stomatal closure. Therefore, RtNAC055 reduced the transpiration rate and increased the WUE and photosynthetic rate of transgenic plants by modulating ROS levels and promoting stomatal closure.

Stomatal movements control plant transpiration and photosynthesis and optimize photosynthetic CO_2_ uptake, thus modulating vegetative growth and biomass accumulation [91]. H_2_O_2_ functions as a secondary messenger in stomatal closure induced by ABA. In addition, MeJA stimulates ROS production via plasma membrane NAD(P)H oxidases [14]. Heterotrimeric G-protein is induced by MeJA, increasing the Ca^2+^ content and H_2_O_2_ accumulation and causing stomatal closure [92]. MeJA induces ROS production and increases the Ca^2+^ level in guard cellsin a manner mediated by COI1, and MPK9 and MPK12 accelerate MeJA-induced stomatal closure by activating S-type anion channels [93]. However, the mechanism by which MeJA affects TFs and regulates ROS production is unclear. MYC2, the master TF in JA signaling, is essential in JA-induced physiological processes. In this study, the expression of JA biosynthesis-related genes (*RtMYC2/AOS1*.*2/AOC4/LOX3/AOS1*.*1*) in leaves was significantly induced in *R*. *trigyna* under drought and *RtNAC055* responded to MeJA but not ABA. ABA contents and ABA signaling pathway genes (*PtNCED3/ABF3/ABI2/PP2C.D2*) activity in OE *RtNAC055* poplar were not significantly different under PEG treatment than in the WT group. Meanwhile, JA accumulation was higher in *RtNAC055* OE callus than WT and RNAi callus. Y1H and dual-luciferase assays confirmed the direct regulatory effect of RtMYC2 on *RtNAC055* expression. Therefore, we speculated that ABA may not involve signaling to regulate RtNAC055 and enhance drought tolerance. MeJA is vital in the response to drought of *R*. *trigyna*. Drought induces JA accumulation in plants, and upregulates *RtMYC2*, an upstream TF of *RtNAC055*. In our study, RtNAC055 promoted ROS accumulation in stomata by regulating the expression of *RtRbohE*, thus modulating stomatal closure. Stomatal closure controls *R*. *trigyna* transpiration, photosynthesis, and water loss, affecting its drought tolerance.

Osmotic stress induces ROS accumulation in guard cells and regulates stomatal movements. ROS accumulation in stomata is regulated by NAC TFs. In *O. sativa*, SNAC1 regulates OsSRO1c, expressed under stress in guard cells. Overexpression of *OsSRO1c* lowers transpiration-mediated water loss by triggering H_2_O_2_ production, thus reducing guard cells and thus diminishing the aperture of open stomata [53]. A banana NAC transcription factor (*MusaSNAC1*) strongly expressed in guard cells under drought accelerated stomatal closure by promoting H_2_O_2_ production in guard cells [54]. In this study, the *RtNAC055* promoter was expressed specifically in root and stomatal guard cells. Y1H and dual-luciferase assays revealed the direct binding of RtNAC055 to an ROS production-related gene (*RtRbohE*). We speculate that RtNAC055 controls stomatal closure by modulating ROS production in guard cells. Indeed, H_2_O_2_ accumulation in stomata was increased in the *RtNAC055*-OE lines, and the ROS production-related gene (*PtRbohD/F*) was enhanced and stomatal closure was accelerated under drought stress. In addition, the transgenic poplar showed enhanced drought tolerance with reduced water loss and ROS accumulation, and increased *F*_v_/*F*_m_, DF, and biomass. Therefore, RtNAC055 controls stomatal closure by modulating the ROS level in stomatal guard cells under drought stress, reducing the transpiration rate and increasing WUE and photosynthesis. NAC TFs also promote ROS accumulation in plant tissues to response to abiotic stress. *GmSIN1*, a salt-induced NAC TF in *Glycine max*, directly activates expression of *GmNCED3*s and *GmRbohB*s to increase the ABA and H_2_O_2_ levels in leaves and roots, resulting in increased salt tolerance and root length [94]. Herein, the H_2_O_2_ content was higher in transgenic poplar leaf and *R*. *trigyna* OE callus than in the WT. Therefore the higher H_2_O_2_ level in OE lines may act as a secondary messenger regulating stomatal closure and maintaining antioxidant system balance to defend against drought stress.

NAC TFs regulate DREB expression to alter drought tolerance in plants [34, 95, 96]. In *Arabidopsis*, DREB1A/DREB2 regulate drought-related gene expression to enhance drought tolerance [97]. DNA demethylation in the DREB2A promoter may enhance drought tolerance and promote root system development in *Malus prunifolia* [98]. MsDREB6.2 enhances the expression of two AQP genes and accelerates stomatal closure and density, which reduces water loss under drought stress [99]. In *Arabidopsis* and *Solanum lycopersicum*, JUNGBRUNNEN1 (JUB1), an NAC TF, improves drought tolerance by directly binding to the promoters of DREB1, DREB2, and DELLA to induce the expression of drought-response genes [100]. In *G. max*, DREB1A is regulated by GmNAC20 and promotes lateral root formation to improve drought tolerance [101]. *CsATAF1*, an NAC TF induced by ABA, improves drought tolerance by activating *CsDREB2C* expression to reduce ROS production [102]. qRT–PCR results showed an upregulation in *RtDREB1*.*1* expression in response to drought treatment and had a similar pattern to RtNAC055. Y1H and dual-luciferase assays showed the direct binding between RtNAC055 and the promoter of *RtDREB1*.*1*. In addition, the expression of the dehydration-responsive gene *PtDREB2*.*2/2*.*6* was higher in *RtNAC055* OE poplar lines. Therefore, RtNAC055 directly regulates members of the DREB family to enhance drought tolerance.

Proline protects against abiotic stresses and oxidative damage and accumulates in plants experiencing environmental stress. Together with its osmolyte function, proline has multiple antioxidant activities [103, 104]. NAC TFs regulate proline accumulation to influence ROS production and modulate antioxidant enzyme activities. GmNAC8 improves SOD activity by altering the proline content to increase drought tolerance. BpNAC012 activates the core *cis*-element CGT[G/A] to promote expression of the proline-synthesis gene *P5CS1*/*2*, increasing antioxidant enzyme activities in *BpNAC012 *OE transgenic birch [74]. AtNAC055 enhances drought tolerance by increasing P5CS1 expression, triggering proline production [71]. Here, *RtP5CS1* expression was significantly increased by drought in *R*. *trigyna* seedlings. RtNAC055 directly bound to the *RtP5CS1* promoter. Moreover, the OE poplar lines accumulated more proline because of upregulation of a proline biosynthesis gene (*PtP5CS2*), compared with WT poplar under drought stress. Therefore, RtNAC055 regulates *P5CS* expression to increase POD and SOD activity to enhance plant drought tolerance.

In conclusion, notably, the *R. trigyna* responed to drought required RtMYC2 and was regulated by MeJA. RtNAC055 directly binds to an ROS production-related gene (*RtRbohE*), leading to greater H_2_O_2_ accumulation in stomata and accelerated stomatal closure. The relationships among *RtNAC05*5, *RtDREB1.1*, and *RtP5CS1* constitute a feed-forward loop responsible for rapid amplification of drought stress signals and maintain higher-level antioxidant enzyme activities. RtNAC055 regulated stomatal closure, reduced the transpiration rate and water loss, and enhanced photosynthetic efficiency and drought resistance by maintaining the oxidant–antioxidant balance in a manner mediated by MeJA (Fig. 10).

## Materials and methods

### Plant materials and treatments

*Reaumuria trigyna* seeds were acquired from Eastern Alxa–Western Ordos, a salinized desert in Inner Mongolia, China. Three normal seedlings (similar dimensions) were cultivated in *in vitro* tubes containing half-strength Hoagland’s medium augmented with 20% PEG6000 (w/v) or 400 mM NaCl (stress induction), and 10 μM ABA or 10 μM MeJA treatment, respectively, for different times (0, 3, 6, 12, and 24 h). Stems/leaves were snap-frozen in liquid nitrogen directly following treatment and kept in storage (−80°C) until analyses.

*Arabidopsis* ecotype Columbia-0 (Col-0) served as WT control. *Arabidopsis* mutant *atnac055* (stock name SALK_011069C) was procured from Arashare. Seeds were sown on half-strength Murashige and Skoog medium (½MS) (48 h/4°C) and then transferred to the culturing room (22°C/16 h:8 h light:dark cycle).

*Populus davidiana* × *P*. *bolleana* plants were grown in WPM. Rooted plantlets were cultured in a glasshouse (25°C/16:8 h light:dark cycle) [62]. To investigate drought stress tolerance, 14-day-old plantlets were transplanted into solid WPM or Hoagland’s medium.

### RNA extraction and qRT–PCR analysis

Total RNA was collected using an Eastep^®^ Super Total RNAExtraction Kit (Promega) according to kit protocols. cDNA was synthesized using a TRANS RT–PCR assay (TRANS). RT–qPCR was conducted on the Rotor-Gene Q^®^ platform (Qiagen) using TransStart^®^ Green qPCR SuperMix (Transgen Biotech). *RtActin1* and *PtActin1* served as internal controls. Relative gene expression was identified using the 2^–ΔΔCt^ method. Supplementary Data Table 2 lists all involved primers.

### Isolation and analysis of *RtNAC055*

*RtNAC055* cDNA corresponding to an ORF was developed by RT–PCR using a TRANS RT–PCR Kit (TRANS). Sequence analysis of RtNAC055 homologs was carried out on NCBI Protein Blast (https://blast.ncbi.nlm.nih.gov/Blast.cgi). Claustal X was used for multiple alignment, and MEGA 5.0 software was used to develop a phylogenetic tree with the neighbor-joining technique. The candidate gene promoter region was amplified using a Universal Genome Walker Kit^®^ 2.0 (Clontech) in line with kit protocols. Elements of *RtNAC055* were analyzed using the PlantCARE (https://bioinformatics.psb.ugent.be/webtools/plantcare/html/) online tool together with the plant *cis*-acting regulatory DNA elements (http://www.dna.affrc.go.jp/PLACE/) database [63, 64].

### Vector construction and genetic transformation

A plant expression vector was constructed by ligating the coding region of *RtNAC055* into pCAMBIA2301-GUS. A 360-bp fragment of *RtNAC055* was introduced into the pK7GWIWG2D vector to generate the RNAi plasmid. The recombinant plasmids were introduced into *Agrobacterium* (EHA105). To probe the *RtNAC055* expression profile, cloned fragments of the *RtNAC055* promoter were inserted in the pORE-R1 vector using a one-step cloning technique.

Transgenic *Populus davidiana* × *P*. *bolleana* poplars were generated using the *Agrobacterium*-mediated method [62]. 35S:RtNAC055 and *RtNAC055*pro:GUS constructs were introduced into *atnac055* (SALK_011069C) mutant and Columbia *Arabidopsis*, respectively, using the floral dip technique [65]. To generate transgenic callus, wild-type *R. trigyna* calli were used as the background for *Agrobacterium*-mediated transformation. The transformed callus was cultured on solid MS medium supplemented with NAA and 6-BA in the dark at 25°C for 3 days and then cultured on solid MS medium with timentin and kanamycin to obtain positive lines; the transformed callus was subcultured every 14 days.

### Subcellular identification/transactivation activity

The full-length (lacking the termination code) *RtNAC055* sequence was cloned into pCAMBIA1300-GFP vector to obtain recombinant plasmids. Vectors (recombinant 35S:GFP:RtNAC055 + control 35S:GFP vectors) were transformed into *Agrobacterium tumefaciens* (GV3101) using electroshock transformation and separately infiltrated into *N*. *benthamiana* leaves through a needle-free syringe [66]. Images were captured after 72 h with a confocal laser scanning microscope (Zeiss 710 Meta^®^, Germany).

The *RtNAC055* coding sequence was inserted into pGBKT7 (Clontech) for generating in-frame fusion onto the GAL4 activation domain, while recombinant plasmids were introduced into yeast AH109 (Supplementary Data Table S1). Transactivation activity was identified using previous protocols [42].

### Yeast one-hybrid/dual-luciferase assessments

To verify binding of RtNAC055 to the NAC recognized sequence (CGT[A/G]) and the *RtRbohE/P5CS1/DREB1*.*1* promoter, full-length RtNAC055 was cloned into pGADT7 to obtain an effector construct. The target sequence (CGT[A/G]) with tandem repeats was placed in pAbAi as reporter vector. The recombinant effector/reporter vectors were co-introduced into Y1H yeast. Transformants were diluted in a 10-fold series, and consequently grown on drop-out medium for 3–5 days at 30°C.

Target gene promoter fragments were cloned into pGreenII 0800:Luc vector, transformed into *A. tumefaciens* (GV3101), and injected, including empty vector pGreenII62-SK or 35S:RtNAC055, into *Nicotiana tabacum* leaves. Samples were visualized using a reduced-light cooled CCD imaging platform (NightSHADE LB 985).

### Physiological measurements and H_2_O_2_ histochemical staining

A SPAD-502 chlorophyll meter (Konica Minolta) was employed to determine plant chlorophyll content. Leaf temperature was measured using a thermal imaging camera. POD, SOD, MDA, PRO, and H_2_O_2_ activities were determined using corresponding assay kits (Keming Bioengineering Institute). The H_2_O_2_ production in guard cells was analyzed by using 50 μM H_2_DCFDA (Coolaber) as described previously [67, 68]. Leaves were incubated (20 min in darkness) in a staining buffer (loading buffer containing 50 μM H_2_DCFDA and 10 mM MES–Tris, pH 6.15), and washed five times in phosphate-buffered saline to remove excess H_2_DCFDA. A fluorescence microscope was used to detect green fluorescence (Eclipse Ci-S; Nikon).

### Photosynthetic index and chlorophyll fluorescence analyses

An infrared gas analytical platform (Li-Cor-6400XT; Li-Cor, USA) was used to probe light curves for RtNAC055 OE and WT poplars, which were well watered for 3 months in a greenhouse. Following the manufacturer’s guidance, we generated light response curves at photosynthetically active radiation (PAR) levels of 0, 10, 20, 50, 80, 100, 150, 200, 400, 600, 800, 1000, 1200, and 1600 μmol/m^2^/s with 400 μmol/mol external CO_2_ [66].

After 30 min of adaptation to darkness, peak PSII quantum yield (*F*_v_/*F*_m_) in leaves from WT and OE lines was automatically monitored and recorded by the Li-Cor 6400XT. A NightSHADE *In Vivo* Imaging System (Berthold Technologies) equipped with a CCD camera was used to measure delayed fluorescence (DF) [69].

### Statistical analysis

Datasets reflected means ± standard deviations. Variation significance was probed using one-way analysis of variance (ANOVA)/Duncan’s multiple range test. Significance for variations across means was evaluated using *P < 0.05 and **P<0.01. All such analyses were conducted using SPSS^®^ 18.0 (SPSS, Inc.).

## Supplementary Material

Web_Material_uhae001
