# Supplementary material for: RtNAC055 promotes drought tolerance via a stomatal closure pathway linked to methyl jasmonate/hydrogen peroxide signaling in Reaumuria trigyna
Source: Hortic Res. 2024 Jan 3;11(2):uhae001. doi: 10.1093/hr/uhae001 (PMC10901477; doi:10.1093/hr/uhae001)
Supplement: Web_Material_uhae001 [file web_material_uhae001.zip › Fig S3.pdf]

A

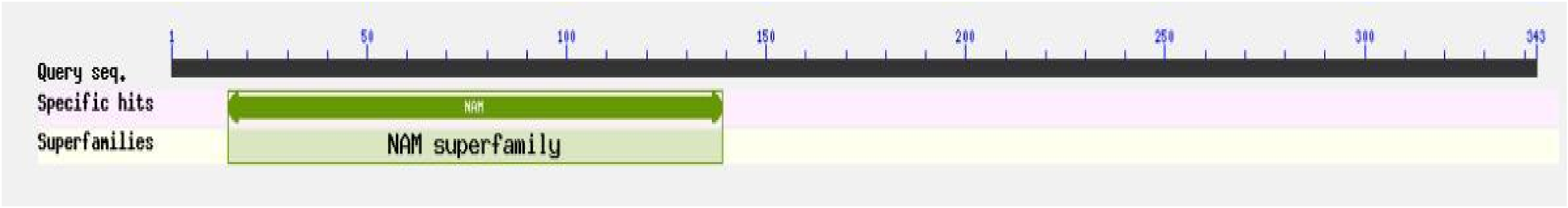
Query seq.
Specific hits
Superfamilies
NAM superfamily

B

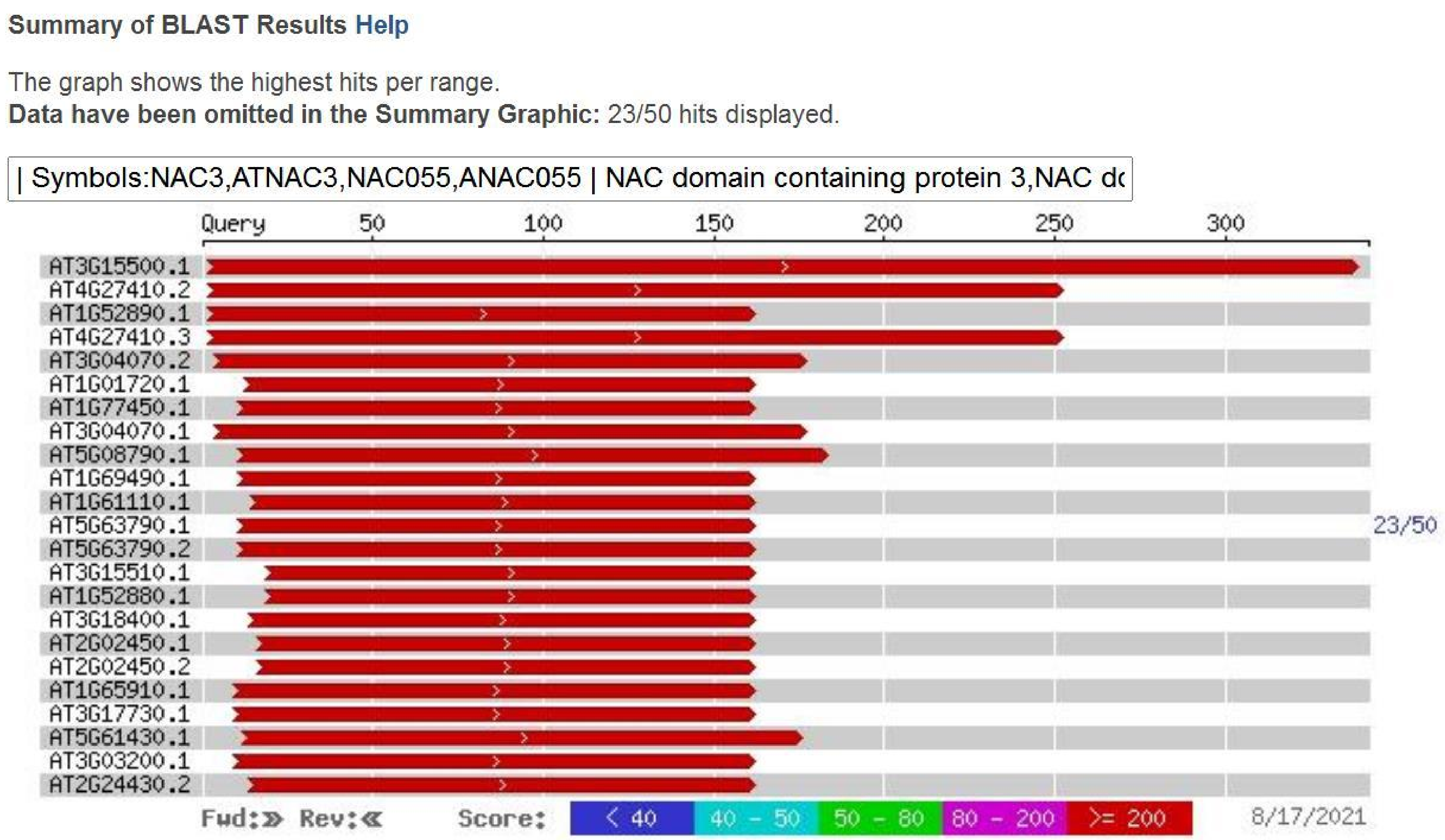
Summary of BLAST Results Help
The graph shows the highest hits per range.
Data have been omitted in the Summary Graphic: 23/50 hits displayed.
| Symbols:NAC3,ATNAC3,NAC055,ANAC055 | NAC domain containing protein 3,NAC dc
Query
50
100
150
200
250
300
AT3G15500.1
AT4G27410.2
AT1G52890.1
AT4G27410.3
AT3G04070.2
AT1G01720.1
AT1G77450.1
AT3G04070.1
AT5G08790.1
AT1G69490.1
AT1G61110.1
AT5G63790.1
AT5G63790.2
AT3G15510.1
AT1G52880.1
AT3G18400.1
AT2G02450.1
AT2G02450.2
AT1G65910.1
AT3G17730.1
AT5G61430.1
AT3G03200.1
AT2G24430.2
23/50
Fwd:» Rev:«
Score:
< 40
40 - 50
50 - 80
80 - 200
>= 200
8/17/2021
