# Supplementary material for: RtNAC055 promotes drought tolerance via a stomatal closure pathway linked to methyl jasmonate/hydrogen peroxide signaling in Reaumuria trigyna
Source: Hortic Res. 2024 Jan 3;11(2):uhae001. doi: 10.1093/hr/uhae001 (PMC10901477; doi:10.1093/hr/uhae001)
Supplement: Web_Material_uhae001 [file web_material_uhae001.zip › Supplementary File-data files.docx]

Table S1

| Motif | Sequence | Function | Distance from ATG |
| --- | --- | --- | --- |
| CAAT-box | CAAT | Common cis-acting element in promoter  and enhancer regions | +940； +568；  +386； +223； |
| CGTCA-motif | CGTCA | MeJA-responsive element | -1033；+229 |
| CAT-box | GCCACT | cis-acting regulatory element related to meristem expression | +258 |
| TATC-box | TATCCCA | Gibberellin-responsive element | +1169 |
| TCCC-motif | TCTCCCT | Part of light responsive element | +1268 |
| TGA-element | AACGAC | Auxin-responsive element | -689 |
| A-box | CCGTCC | cis-acting regulatory element | +974 |
| ABRE | ACGTG | Abscisic acid-responsive element | +1063；+284 |
| ACE | GACACGTACG | light responsive element | -1060 |
| LTR | CCGAAA | Low temperature responsive element | +1163 |
| G-box | TACGTG | light responsive element | -283 |
| GA-motif | ATAGATAA | light responsive element | -724；-658 |
| GC-motif | CCCCCG | Enhancer-like element involved in anoxic specific inducibility | -979 |
| MBS | CAACCA | MYB binding site involved in drought-inducibility | +202；+1292 |

Table S2

| **RtNAC055-ORF-F:** | 5’-ATGGGTTTAGCCGAGAGAGATC-3’ |
| --- | --- |
| **RtNAC055-ORF-R:** | 5’-TCATCGCTGCCTGAACCCGTA-3’ |
| **RtNAC055-qRT-F:** | 5’-ATTTCATTGCCGTCGCTTCG-3’' |
| **RtNAC055-qRT-R:** | 5’-CTGAACCCGTACCCGTTTGA-3’ |
| **RtNAC055-Sac I-F:** | 5’-GCGAGCTC ATGGGTTTAGCCGAGA-3’ |
| **RtNAC055-Xba I-R:** | 5’-GCTCTAGA TCGCTGCCTGAACCCG-3’ |
| **RtNAC055-Sma Ι-F:** | 5’-GCCCCGGGATGGGTTTAGCCGAGA-3’ |
| **RtNAC055-Pst Ι-R:** | 5’-GCCTGCAGTCATCGCTGCCTGAA-3’ |
| **RtNAC055-GSP1：** | 5’-CGGCGGAACTCTGGGTATGAGCGAACTGA-3’ |
| **RtNAC055-GSP2：** | 5’-ATCCACCATAGCCGCCACCAGTGAATGGT-3’ |
| **RtP5CS1-GSP1:** | 5’-AGACAAGTCCAGCGTTCGCAACACCAATGC-3’ |
| **RtP5CS1-GSP2:** | 5’-CTGGTTTTCCAAGGGCCTTTTCCTGCACCA-3’ |
| **RtDREB1.1-GSP1:** | 5’-TGGCATCACCACCTCCTGATGGCAAACTG-3’ |
| **RtDREB1.1- GSP2:** | 5’-TGGCATCACCACCTCCTGATGGCAAACTG-3’ |
| **RtNAC055-F:** | 5’-CTCTCTCAAGCTTGGATCC(BamHΙ)ATGGGTTTAGCCGAGAGA-3’ |
| **RtNAC055-R:** | 5’-GGCATGCCTGCAGGTCGAC(Sal Ι)TCATCGCTGCCTGAACCC-3’ |
| **proRtNAC055-F：** | 5’-TTCTAGAAGGCCTTGGATCC(BamH Ι)GCTGTTATCTAGCGGGAGTGG-3’ |
| **proRtNAC055-R：** | 5’-CTACAGGACGTAACATGAGCTC(Sac I)GCTACTTTCCGGCATAGA-3’ |
| **qRT-RtACTIN-RT-F:** | 5'-GGAATCCACGAGACCACCTACA-3'; |
| **qRT-RtACTIN-RT-R:** | 5'-GATTGATCCTCCGATCCAGACA-3' |
| **qRT-RtAOS1.1-F** | 5'-GGTCCTGAAGTGCTAAACAAAG-3'; |
| **qRT-RtAOS1.1-R** | 5'-ACTGGGCACGAAATCACCAA-3' |
| **qRT-RtLOX3-F** | 5'-GCTGCATACGGGTAGTCCTC-3' |
| **qRT-RtLOX3-R** | 5'-TGGCTCGCCAGAACTTGATT-3' |
| **qRT-RtLOX1-F** | 5'-GCATATGCACGGGCTACTCT-3' |
| **qRT-RtLOX1-R** | 5'-GACCAGATGTCGAGTCCGTC-3' |
| **qRT-RtAOC4-F** | 5'-GCTTCAACATTGGGCGATGG-3' |
| **qRT-RtAOC4-R** | 5'-AGCTGTTACCGGTGGTTCTG-3' |
| **qRT-RtAOS1.2-F** | 5'-AACCACGCTCGTGCTAAGAA-3' |
| **qRT-RtAOS1.2-R** | 5'-CTCTCTCGTTTCTCGACCCG-3' |
| **qRT-RtMYC2-F** | 5'-GATCAGTGAGCCAGCCTTGT-3' |
| **qRT-RtMYC2-R** | 5'-GTGAGTCGGTTGGTGGAGTT-3' |
| **qRT-RtJAM2-F** | 5'-CACGGTTCGTCCTCACTTCA-3' |
| **qRT-RtJAM2-R** | 5'-CCTGTAAGCTGTTTCCCGGT-3' |
| **qRT-RtRbohE-F** | 5'-TGAGGGCCTTACCGGCATTG-3' |
| **qRT-RtRbohE-R** | 5'-GGCATTGTACCCAGCTAGGCG-3' |
| **qRT-PtDREB2.2-F** | 5'-GGTGGGCATTGCGAAGTTTT-3' |
| **qRT-PtDREB2.2-R** | 5'-TCTGGCGCTTTGAGCCAATA-3' |
| **qRT-PtDREB2.6-F** | 5'-AGAGAACTTGGGGCAAGTGG-3' |
| **qRT-PtDREB2.6-R** | 5'-AGCTACCCAACTGATCCCCT-3' |
| **qRT-PtP5CS2-F** | 5'-TCGCACTGAAGGCGTTACTT-3' |
| **qRT-PtP5CS2-R** | 5'-GAGCCCCATCGCAGAATCTT-3' |
| **qRT-PtNCED3-F** | 5'-AACAGCAGTGCCTTCAACCT-3' |
| **qRT-PtNCED3-R** | 5'-TGTTGCTTTTGGGGCACAAC-3' |
| **qRT- PtABF3-F** | 5'-**ACTGCCGAAGAGACTCAAGC**-3' |
| **qRT- PtABF3-R** | 5'-ACTCCCATCTTCAGCACCAC-3' |
| **qRT-PtPP2C3-F** | 5'-CTGGCCAGTGATGGCCTATG-3' |
| **qRT-PtPP2C3-R** | 5'-AGAAGCTGTTGCGCCTTTTG-3' |
| **qRT-PtPP2.D2-F** | 5'-GTCGGCAACACTGGAGGTAT-3' |
| **qRT-PtPP2.D2-R** | 5'-GGTTCCCTGTTGAATCGTGC-3' |
| **qRT-PtABI2-F** | 5'-CCTCATTCTGGCCAGTGATG-3 |
| **qRT-PtABI2 -R** | 5'-GTTTGAGAGGTACTCAGCTG-3 |

>RtNAC055 Promoter

+1246 CTGTTATCTA GCGGGAGTGG AAACTTAATA ATCCAGGCTG AACGGACAAT TCGGATGTCC GCTACAAGAC

-1246 GACAATAGAT CGCCCTCACC TTTGAATTAT TAGGTCCGAC TTGCCTGTTA AGCCTACAGG CGATGTTCTG

+1176 CCAAACATAA TACCCAAGCA AGCTATTTGT GGAATCTACA TACGTCCACA AGAAACTCAA CCATTAAGTA

-1176 GGTTTGTATT ATGGGTTCGT TCGATAAACA CCTTAGATGT ATGCAGGTGT TCTTTGAGTT GGTAATTCAT

**G-Box G-Box**

+1106 ACAACCCTCA AATCCGTCAT CTCGTGATAT AGATCAAACA TGGGCCACTT GTTTTGTAGT ATGACTTGAA

-1106 TGTTGGGAGT TTAGGCAGTA GAGCACTATA TCTAGTTTGT ACCCGGTGAA CAAAACATCA TACTGAACTT

**G-Box**

+1036 CGTGGATTAA TAGCAGGTGA ATCCAAGACT TTCACCACTA CACCACAACG AGATCCATAA TTTTTACCGG

-1036 GCACCTAATT ATCGTCCACT TAGGTTCTGA AAGTGGTGAT GTGGTGTTGC TCTAGGTATT AAAAATGGCC

+966  TGATACAATC TTTATTGTCA AACACTTTTC ACAAATAAGT ATATTGACAA ACACTATTAA AAAAAATAAG

-966  ACTATGTTAG AAATAACAGT TTGTGAAAAG TGTTTATTCA TATAACTGTT TGTGATAATT TTTTTTATTC

+896  CATATATTTT ACCACTACCA TTTTAGTTTT TAAGTATCAT TTCAACTAAT TGTATATCCA TTCTAATGGG

-896  GTATATAAAA TGGTGATGGT AAAATCAAAA ATTCATAGTA AAGTTGATTA ACATATAGGT AAGATTACCC

+826  TGTCATTGAA CACTTATTAA AAATTTAATA ATTTACTTAA AATAATATAA TTAAAACATT AATAATAAAA

-826  ACAGTAACTT GTGAATAATT TTTAAATTAT TAAATGAATT TTATTATATT AATTTTGTAA TTATTATTTT

+756  ATACAAATAA ATAAATATAT TAATTATTAA ATTTTCTTTA CAATTTTAAT AAAAATAACA AGTGCTCTTA

-756  TATGTTTATT TATTTATATA ATTAATAATT TAAAAGAAAT GTTAAAATTA TTTTTATTGT TCACGAGAAT

+686  AGGCATGTTT GTCAATCTAT CCATTATCTA TATTATCTAA TAATATCACT TAATGTCGTT TATTTTTACT

-686  TCCGTACAAA CAGTTAGATA GGTAATAGAT ATAATAGATT ATTATAGTGA ATTACAGCAA ATAAAAATGA

+616  TACTATTATA TTATTTATAT TATCTATCAT TATTACTTTA TAAGTAGAAA CTATCACTTT TAATATAATA

-616  ATGATAATAT AATAAATATA ATAGATAGTA ATAATGAAAT ATTCATCTTT GATAGTGAAA ATTATATTAT

+546  TCTAAAATAT TCTATATCAT TTACTGTTAT TAACTCATAA AATAATATAA AAATATTATA CTATTAAAAA

-546  AGATTTTATA AGATATAGTA AATGACAATA ATTGAGTATT TTATTATATT TTTATAATAT GATAATTTTT

+476  ATATATATAT AAATATCAAT ACTTATTATT TTAAATAAAA AAATTATTAA ATACTATTAT TACTTTAAAT

-476  TATATATATA TTTATAGTTA TGAATAATAA AATTTATTTT TTTAATAATT TATGATAATA ATGAAATTTA

+406  CAATTATTAT TTTAAAATAT CATTTCAAAT AAATTCAGAC CATGATTGTC GTCGAACACC CGTCCGGGGG

-406  GTTAATAATA AAATTTTATA GTAAAGTTTA TTTAAGTCTG GTACTAACAG CAGCTTGTGG GCAGGCCCCC

+336  CACTTTACAA AATATCTTTT ACCATATTAT GGGTCTGGCT TCGTTAAGTG ACGTTGACAA GCTCAAGCCA

-336  GTGAAATGTT TTATAGAAAA TGGTATAATA CCCAGACCGA AGCAATTCAC TGCAACTGTT CGAGTTCGGT

**G-Box**

+266  TACTTTATAC GTGTCCGCGT TTTAGGCGAT AACTACTGTA GGACCCACTA TTTTTGTCTA TCTATTGACT

-266  ATGAAATATG CACAGGCGCA AAATCCGCTA TTGATGACAT CCTGGGTGAT AAAAACAGAT AGATAACTGA

+196  GGCGGCTCTG TTTCACTCTC GTCCTCCGCC TATATAAACC GAAATATCCC ACAACAAGCT TGTTTCATTC

-196  CCGCCGAGAC AAAGTGAGAG CAGGAGGCGG ATATATTTGG CTTTATAGGG TGTTGTTCGA ACAAAGTAAG

+126  CTTCTTGCAA TTGATATTTT ATTTACTCGT AAATTGATAG AAAAAGACCA TACTCTTCTC AACTCTCAAT

-126  GAAGAACGTT AACTATAAAA TAAATGAGCA TTTAACTATC TTTTTCTGGT ATGAGAAGAG TTGAGAGTTA

+56  CTCTCTCCCT TGTAGAGAAG GAAAATTCAA CAGTAGACGG AAATTCCTTA AAATGG

-56   GAGAGAGGGA ACATCTCTTC CTTTTAAGTT GTCATCTGCC TTTAAGGAAT TTTACC

>RtP5CS1 Promoter

+937 CCATAACTAA GCTGCTAAGT ATTCCCTTCG AAGTGTTTGG TAATACAAAC TGTTTAACTC GCTGAAATAT

-937 GGTATTGATT CGACGATTCA TAAGGGAAGC TTCACAAACC ATTATGTTTG ACAAATTGAG CGACTTTATA

+867 ATAAAATGAC ATAAAAGGAT ATGGTTATAT TAATTATTGA TGATTTAATG AGAGGGTATA TGTGGAGAAA

-867 TATTTTACTG TATTTTCCTA TACCAATATA ATTAATAACT ACTAAATTAC TCTCCCATAT ACACCTCTTT

+797 AAAAATAAAC TCAAACAAAT AAGCTCCTAC AAATAAACTA AAAGCTACTT GAGAAAGCTT TTTAGAAAAG

-797 TTTTTATTTG AGTTTGTTTA TTCGAGGATG TTTATTTGAT TTTCGATGAA CTCTTTCGAA AAATCTTTTC

+727 AGTTTTTTTC TTTAAAAAAA CTACTTACCA AACGGAACTA TTTCATACTA AGGAGCTTAT TTTAATAAGC

-727 TCAAAAAAAG AAATTTTTTT GATGAATGGT TTGCCTTGAT AAAGTATGAT TCCTCGAATA AAATTATTCG

+657 TAAAAGCTAA AAGCTCTCAT TTAAGCTACT AAAAAGCCAT GCCAAACGCA CTCTTAATTT TTGTATTCAA

-657 ATTTTCGATT TTCGAGAGTA AATTCGATGA TTTTTCGGTA CGGTTTGCGT GAGAATTAAA AACATAAGTT

+587 AACTAAAATG ATAGTATTTA TTTGAGAACA GAGAATGTTT TAATATTATG AAATTATGAA AATTGCAAAG

-587 TTGATTTTAC TATCATAAAT AAACTCTTGT CTCTTACAAA ATTATAATAC TTTAATACTT TTAACGTTTC

+517 GAGGGAGCAG CAACATATAT AATTTATCCG CTAGCTTGCT GCTATTAGAC ACGCTCAGGT GAGTGGAGGG

-517 CTCCCTCGTC GTTGTATATA TTAAATAGGC GATCGAACGA CGATAATCTG TGCGAGTCCA CTCACCTCCC

+447 CTGAATTTTA GATCAAATAA TCACAATTTG TTTTCCGAGC TCTAATAACT CCTGTAAGCA TGAGATGAGA

-447 GACTTAAAAT CTAGTTTATT AGTGTTAAAC AAAAGGCTCG AGATTATTGA GGACATTCGT ACTCTACTCT

+377 TATTTCAGCC TTTCACGTAT CTCGTGTGTG GCTATCCGTG ACTTGGAAGA TTTTTCTTAG AAAGAGGAAG

-377 ATAAAGTCGG AAAGTGCATA GAGCACACAC CGATAGGCAC TGAACCTTCT AAAAAGAATC TTTCTCCTTC

+307 GGAAATTAAT TAAAAAAAAA TACTAATAAA ATAAATAAGT ATTTGTCCGT TTACGGCCTC AGGGCCGCAT

-307 CCTTTAATTA ATTTTTTTTT ATGATTATTT TATTTATTCA TAAACAGGCA AATGCCGGAG TCCCGGCGTA

+237 TTAAGGCGGC CGGAGAGAGT GATTGGGGAG ATACAAATTT GACGTGGGAG CGGAGTCCGT TGCTGCCCTA

-237 AATTCCGCCG GCCTCTCTCA CTAACCCCTC TATGTTTAAA CTGCACCCTC GCCTCAGGCA ACGACGGGAT

+167 CCAGTACCAC TCAGTCGTCA CCGAGTCTCC TCATCTTCCA TTGAGAAGTG TGAAGGGGGA AAAAAGAAGA

-167 GGTCATGGTG AGTCAGCAGT GGCTCAGAGG AGTAGAAGGT AACTCTTCAC ACTTCCCCCT TTTTTCTTCT

+97 AAAAAAAAAA AGAAAAAACA GAAACATACA CTTACTGAGG TGATAGGGTG GCATTTCTGT TTCGTTATCC

-97 TTTTTTTTTT TCTTTTTTGT CTTTGTATGT GAATGACTCC ACTATCCCAC CGTAAAGACA AAGCAATAGG

+27 CTTCCCCCTG AATCATCAAT CATGGAC

-27 GAAGGGGGAC TTAGTAGTTA GTACCTG

>RtDREB1.1 Promoter

+1215 GCTGATCTTT CTCTTTTCTT CCGTTCGTCT CAGACTTCTT TTTATCCGAA AGGAGGACGT CGGCGATCGG

-1215 CGACTAGAAA GAGAAAAGAA GGCAAGCAGA GTCTGAAGAA AAATAGGCTT TCCTCCTGCA GCCGCTAGCC

+1145 TTCCTAGCTT CAGTCCTTGA GTGCGAAGTT GAGTCTACTT CAGCCAAAAA CATCACAAAA TTCCTAATCA

-1145 AAGGATCGAA GTCAGGAACT CACGCTTCAA CTCAGATGAA GTCGGTTTTT GTAGTGTTTT AAGGATTAGT

+1075 CCCGAATTCC GGCGATGTCT TCAGCGTCCT CAGATCGGTA TGTGTCTACT TTTATAGTTC GTTTCTTTTT

-1075 GGGCTTAAGG CCGCTACAGA AGTCGCAGGA GTCTAGCCAT ACACAGATGA AAATATCAAG CAAAGAAAAA

+1005 TCTTTTCTGG TTGATTTCAC TTCTTCGATG GTTCTTCTTG TTTCCAGTGG CGTAATATCC GGGGCAATCG

-1005 AGAAAAGACC AACTAAAGTG AAGAAGCTAC CAAGAAGAAC AAAGGTCACC GCATTATAGG CCCCGTTAGC

+935 TTGTTTTGCT TGTAATTCTT TTCTCAATTG TGAATTATTC GTACATTGTC CTGTTTGTGC TTGTGCTTTA

-935 AACAAAACGA ACATTAAGAA AAGAGTTAAC ACTTAATAAG CATGTAACAG GACAAACACG AACACGAAAT

+865 ATTCCGAAAC TAACGTGGTT TATCGACTTG TTGAACTCGG TCCTTTGGTT TGATTTAGAT TTTGGGAGTT

-865 TAAGGCTTTG ATTGCACCAA ATAGCTGAAC AACTTGAGCC AGGAAACCAA ACTAAATCTA AAACCCTCAA

+795 TGGAAAATTG ATTACGGTGT TTTTCTGAAT TTCACTGTGT TGAATGTTTG ATTGGTATAG GTTTAGAGGT

-795 ACCTTTTAAC TAATGCCACA AAAAGACTTA AAGTGACACA ACTTACAAAC TAACCATATC CAAATCTCCA

+725 TCCTATTAGG GTTTTTGTTG AATCTTGATT ATTTTTGCTG TTTGGCGGGG TTACGCAAGT TAGGTTTCGT

-725 AGGATAATCC CAAAAACAAC TTAGAACTAA TAAAAACGAC AAACCGCCCC AATGCGTTCA ATCCAAAGCA

+655 AATAGATTTG GGATTTTTTA TGCTTCCGAA GTTCGGTCAC CTGGTGTGCT GTTCTTTCCT TCTTCCCATT

-655 TTATCTAAAC CCTAAAAAAT ACGAAGGCTT CAAGCCAGTG GACCACACGA CAAGAAAGGA AGAAGGGTAA

+585 GAGTTTACGT TTGATTTTGA TTTGGATGTG TCGTCTAGTA GGGGATTTTG TTTTACAATC GAAAGTATCG

-58 CTCAAATGCA AACTAAAACT AAACCTACAC AGCAGATCAT CCCCTAAAAC AAAATGTTAG CTTTCATAGC

+515 TTCTTGAATT AGCGGCTAGA TATTCGACAA TCCAATTCCA AAGATTTTGT TCTCGTCCTA TCTCAAGTTG

-515 AAGAACTTAA TCGCCGATCT ATAAGCTGTT AGGTTAAGGT TTCTAAAACA AGAGCAGGAT AGAGTTCAAC

+445 CGTTAACAAT TAAGATGAGG AACTTTCAGG CTTAGTTTTA CTCCGTTTAT GTCTATAATG TAATAAAACC

-445 GCAATTGTTA ATTCTACTCC TTGAAAGTCC GAATCAAAAT GAGGCAAATA CAGATATTAC ATTATTTTGG

+375 TTTTGTTCAT TCCTGTTGCA GATGGATCGT CTCTGTTCTC TGAATTCTTG CGAACGAGTG GTGCTTGGTT

-375 AAAACAAGTA AGGACAACGT CTACCTAGCA GAGACAAGAG ACTTAAGAAC GCTTGCTCAC CACGAACCAA

+305 GGCTGTGAAC CATCCGTCTA ACTATACTTC TTTTCCTGTC TTTCTTGCTC TTTAATTTTT CAGGTTAGGC

-305 CCGACACTTG GTAGGCAGAT TGATATGAAG AAAAGGACAG AAAGAACGAG AAATTAAAAA GTCCAATCCG

+235 AGTCATGACC GTGCTGCTTC TACCCAGAAT GGTGTACCCT GTGATTACAC CAAGAAACGT AGGCCTCGAG

-235 TCAGTACTGG CACGACGAAG ATGGGTCTTA CCACATGGGA CACTAATGTG GTTCTTTGCA TCCGGAGCTC

+165 GTGATGGAAA AGCTGTTGCA GAAACCATTG CTAAATGGAA GGAGATCAAC AACAATCTTA CTTCTGCTGC

-165 CACTACCTTT TCGACAACGT CTTTGGTAAC GATTTACCTT CCTCTAGTTG TTGTTAGAAT GAAGACGACG

+95 TGTCATAAGC AAACCACCTG CCAAGGGATC TAAGAAAGGT TGTATGAAGG GTAAAGGTGG ACCTGAGAAT

-95 ACAGTATTCG TTTGGTGGAC GGTTCCCTAG ATTCTTTCCA ACATACTTCC CATTTCCACC TGGACTCTTA

+25 CAGCGCATCA ACTATTCGGG GGAAT

-25 GTCGCGTAGT TGATAAGCCC CCTTA

>RtRHOBE Promoter

TTACTATAGGGCAACGCGTGGTCGACGGCCCGGGCTGGTTCTCTCTCTCCCTCCTTCGTTAATCTCCAGTAAAACAGAGTACATCTCCTGTCAGAAGAAATTCAAAATGCGGTCGTCTTCGTCGTTCGGCAGCGGCTCAAGAAGATCGAATTACAGTCGTCAAATGGATTTAGCCGAGGAAGATCTTGCAGTAGCAGCAGAATACGGTTTAGGTGGAGCAATGTTACCGATTTTTCTAAACGATATACGAGCTTCACAACACGACAACAGTTATAATAATCAAGAGCTTGTTGAAGTTGAATTAGAATTAGATGATAATAACATTACACTTTGTAGTGTAACACCAACGGCAACCATGACACCAACACCTAATCGACTTAATTTTCCAGATGATCATCATACTACATTTGCTGGAAAATTAACGAGAAGTGTATCGAATACGTCCCATAAATTGAGGAATAAGTTAGGTTGGTGGTTGGCAAGATCTACATCGTGGAAATCATCGGCGTCATCGGTAGTTGATGTGGAACAGCCAGCTGGTGGTGGTGGTGCGACGGTAAGTACGCCGAAGCTGGATGCGTTGAGACTTAGGAGGGAACAATTACAGAGGACGGGATCGAGTGCACAGAGAGCGCTTCGTGGACTTAGGTTTATTAGTAAGACGACGGCGGCGGGAACGAAGGGTTCGGCGACGGCGGATGCTGGACAGCAGTTATGAAGCAGGTTAGGATAGGTTTGATGAGTTGCAAGTGGATGGATTGGTGTCGAGGGAACAATTCGGTGAATGCATAGGTCAGTTAGTAGGGAAAATAGTATTATGATGGATGATATTTTAATCTAATTAGTGAAATTAAATTAAATAAAAACATGAATGAATGCAGGGATGAGAGATTCCAAGGAATTTGCCGAAGGGATATTTGATGCGTTAGCGAGAAGGAAGAGACAGAGGATGAGTAAGATTTCAAAAGAGGAACTGTACGATTTTTGGCTTCAACTTTCCGATCAAAGTTTTGACGCCCGTCTTCAGATTTTCTTCGACATGTAATTTTCTTCTCTCTCATGTCTTATTTCCACTCTTTTTTATTTTTTGCTTCAAAACTGACGTTACTTTTATTTTTCCAGCGCTTTAAATTTCTCTTGTTTCGATTTTTATTTTTATTTTTATTTTTGGTTTCCCATATCGGACGGAGATCGTATTTTTTATTTTGTTTTTGCTCTTTCTTGCAAAAATGCTATTTTCCTGTTTATTTAGATTATGCCTAATTAAAGATGCATTTTAACTAATTAACAATTTTCCGTATCGACTTGCTTTGTAACCGATGTCAGCTAGCAGTTTACCCACTTATTTCTACTGCTTCTCACGTCTTTTCCAAACATCTGCTCCTTTCATGTTCCTATTTTGATTTCTTGGATACTACTTGTGTGAATACCACTGATCGTGAATAACTACTAATTTGGTAGGTTAGCGTCTGCTAACTTCCTTATCGGTAGTTACGATTGCAAATGTTCAGGTTATGCAATTCCTTTTTCTCCCTCCGTCTCTATCTATCTATTTAGAAATAGTCAAGCTTTAAACGTGCTTCTTTTACGTCAGGGCTGATCGTAATGCAGATGGAAGAATTACTAGAAAGGAAGTTCAAGAGCTCATTATGCTCAGTGCTTCAGAATCTCTGGAAGATCCGCGCGTACCGAGTTCTAATTCACTGGCCGTCGTTTTACAACGTCGTGACTGGGAAAACCCTGGCGTT

>RtNAC055

ATGGGTTTAGCCGAGAGAGATCCGCTGGCTCAATTGAGCTTACCTCCTGGATTCCGATTCTATCCAACAG

ATGAAGAATTAATGGTTCAGTATCTATGCCGGAAAGTAGCCGGCCATGAATTCCCACTTCAAATTATTGG

CGATATTGATTTGTACAAATTCGACCCATGGGTCTTACCAAGTCTGGCGACGTTCGGGGAAAAAGAATGG

TATTTTTTTAGTCCAAGAGACAGAAAATATCCAAACGGGTCAAGACCTAACAGAGTAGCCGGATCGGGAT

ATTGGAAAGCAACCGGAACAGATAAGATCATTACGACGCAAGGAAGAAAAGTAGGGATAAAGAAAGCTTT

GGTGTTTTATATTGGAAAAGCTCCAAAAGGTACCAAGACTAATTGGATCATGCATGAATATCGTCTCTCC

GATCCTCAAAGGAAAAACGGCAGCTCCAAGTTGGATGAATGGGTATTATGTAGAATCTATAAGAAAAGCA

GCTCATCGTCATCAACAAAGCAGCTACCTATCATTTCATCACCAAACTGCACATCTATGTCATCACCATT

CACTGGTGGCGGCTATGGTGGATCCTCCCCATCTTCCTCCTCTCACATCGGCAGCTTTCTTGAAGCGATG

CCGGACCAATTGTCTGATCACGATGTCTCGCTGAAGACGGATGATAAATTGGGTGATCTCTCAAAACTCG

GGTCCGGTAATTTTGACTGGGCAACAGTACTTGGGTTAACAACTACCCCTGAACTCAATTCTCATATACA

ACAAGGAAGAACGGGACAACAGCAGCAGATACAACAATCTCCAGCGGCAGCTCAAGGGAATAACAATTTC

TCTGCTATTTCATTGCCGTCGCTTCGACAGGTCGACGACGAGGTTCAGAGCAGAGTCAAGAATTCTCATC

GAGTTAACAATAACAATCAGTTCGCTCATACCCAGAGTTCCGCCGGCATGATTGACCAGTTTGGTATTAT

TCAGTATCCTCCTAATCAATCAAACGGGTACGGGTTCAGGCAGCGATGA

>RtMYC2

ATGAATGTCTGGTCAGACGATAACGGGTATGGGATGGATACCTTCATGTCATTCGATTACCCTTCTTCCGCTTCTGTCCCCGCAACCGTCGGTGTTGGTCTTGGTGGCGGAATTTGGCCTCCCTCTTCTTCCCAATCACCTCAACCTTTTAATTTCCCACCTGACTGCTCAGGGAGACCCTTTTTGCAGCCAAACTCTTCTTCCTCCAATCACCAGCCGTCATCCTCCTCTATGGCCGTCTTCAACCAGGATTCACTTCAACAACGTCTCCAGGCTTTAATCGACGGTGCTAGAGAGAGCTGGACTTACGCAATTTTCTGGCAATCCAGTCCCGATCTGGACGTTGATTCAACGTTAATTTGGGGAGATGGGTATTATAAAGGGGAAGAAAATAAAGATAAAAGTAAGAACAAAAGTCTCGATCCCGTCGAACAAGATCACCGGAAAAAGGTATTAAGGGAACTCAACTCCTTGATTTCCGGTAACACTGCATCGCCGGATGATGCAGTCGACGAAGACGTCACCGATACAGAGTGGTTTTTCCTTGTTTCTATGACCCAATCGTTTGCTAACGGTGTCGATTTGCCGGGTCATGCTTTTACGGGATTAAATCCAATTTGGGCTTCTGGGTTAGAGAGATTATCCAGCTCTCCTTTCGATAGGGCGAGGCAGGGATCGGAGTTTGGCCTTCGTACGTTGGTATGTATACCTATACAAGGTGGGGTTGTTGAAATGGGTTCAACTGATGTGATCATCCATAGCTCAGATTTGATGAATAAGTTTAGGATACTATTCAATTACGGGGGCGGAGATGCGGTCAGTAATTGTTCCAATTCAGCAGCACCGGCTGCAGTGCCTGTGCTTGCTCAACCTCACCCTCAACCTTCGGGGGTGCCTCCTGATCAAGGCGAGAATGACCCGTCTGCACTCTGGATCAGTGAGCCAGCCTTGTCGGGTGCTGATGTCAAGAATGGTATTAACGGAGGAGGAGGGTCGAGTTCAAATCTTATTGGTTTGAGTTCGAATCCAATTAAGGACAGTAGTCGTCATGAGCAGCACCAGCAACAGAAAGGGGTAATTCATTTAGATGTTCAGAGCACAAGTGGTGTAAGTGAAAACCCAATTGGAATAAATGTAAACAAACTCCACCAACCGACTCACCTTCAACATCAACCACCAGGGTTTCTCGGTAGGGAATTGAATTTCTCTGATTATGGTTACGGTGGTGGCGATGGGATTGGTTCCAGGAACAACGGGACTATGAATCCCATGAAACCTGAAGCAGGTGAACTCTTATGTTTTGGAGATAATAAGAGGGGAACTTCATGTACAGGTTCTGGAACAATATTTGGTGGTTCCAATTCACAGATGATGGCAGAAGAGTACAACAAAAAGCAGAAAAAGGCGTCACCGACTTCAAGGGGCAGCAACGAAGAAGGGATGATGTCTTTCACTTCAGGCGTCCTCTTACCATCTTCTGGTGTTGTTAAGTCCAGCACCGCAGATTCAGACCACTCGGACCTTGAAGCCTCAGTAAAGGAAGCAGACAGCGCAAGCAGAGTAGTTGACCCTGCAGAGAAAAGACCTCGAAAGAGAGGTCGAAAACCAGCAAATGGTAGAGAAGAGCCACTCAACCATGTAGAAGCAGAGAGACAAAGAAGAGAGAAACTAAATCAGAGATTCTATGCCTTAAGAGCTGTTGTCCCAAACGTATCTAAAATGGATAAGGCATCACTCCTTGGTGATGCTATTTCCTACATAAACGAGATGAAGTCCAAGCTTCAAACTAAGGAATCTGATATTGAAGCATTGCAGAACCAAGTTGATACATTGAAAAGGGAACTAGCTGGCAGAAGAGGAGGATCAGCGTACTCTAATGGACAACAGCCTCCATCTTCCGGCCACCATGATGTTAAGATGTTGGGTGATAAACTTATAGATGTGGAGATTGATGTCAAGATCATCGGATGGGATGCAATGATTCGTGTAACTTCTGGTAAGAAAAACCACCCAGCTGCTAAGCTGATGGTGTCATTAAGAGAGTTGGATTTAGATGTGTGTCATGCTAGTGTGTCGGTGGTTAATGAATGGATGATCCAACAAGCTACCGTGAAGATGGGGAACAGGTATTACAGTGAAGAGCAGCTGAGAATAACACTGGCAGCTAAGATTGCTGATCCCAGATAG
