# Supplementary figures and images for: RtNAC055 promotes drought tolerance via a stomatal closure pathway linked to methyl jasmonate/hydrogen peroxide signaling in Reaumuria trigyna
Source: Hortic Res. 2024 Jan 3;11(2):uhae001. doi: 10.1093/hr/uhae001 (PMC10901477; doi:10.1093/hr/uhae001)

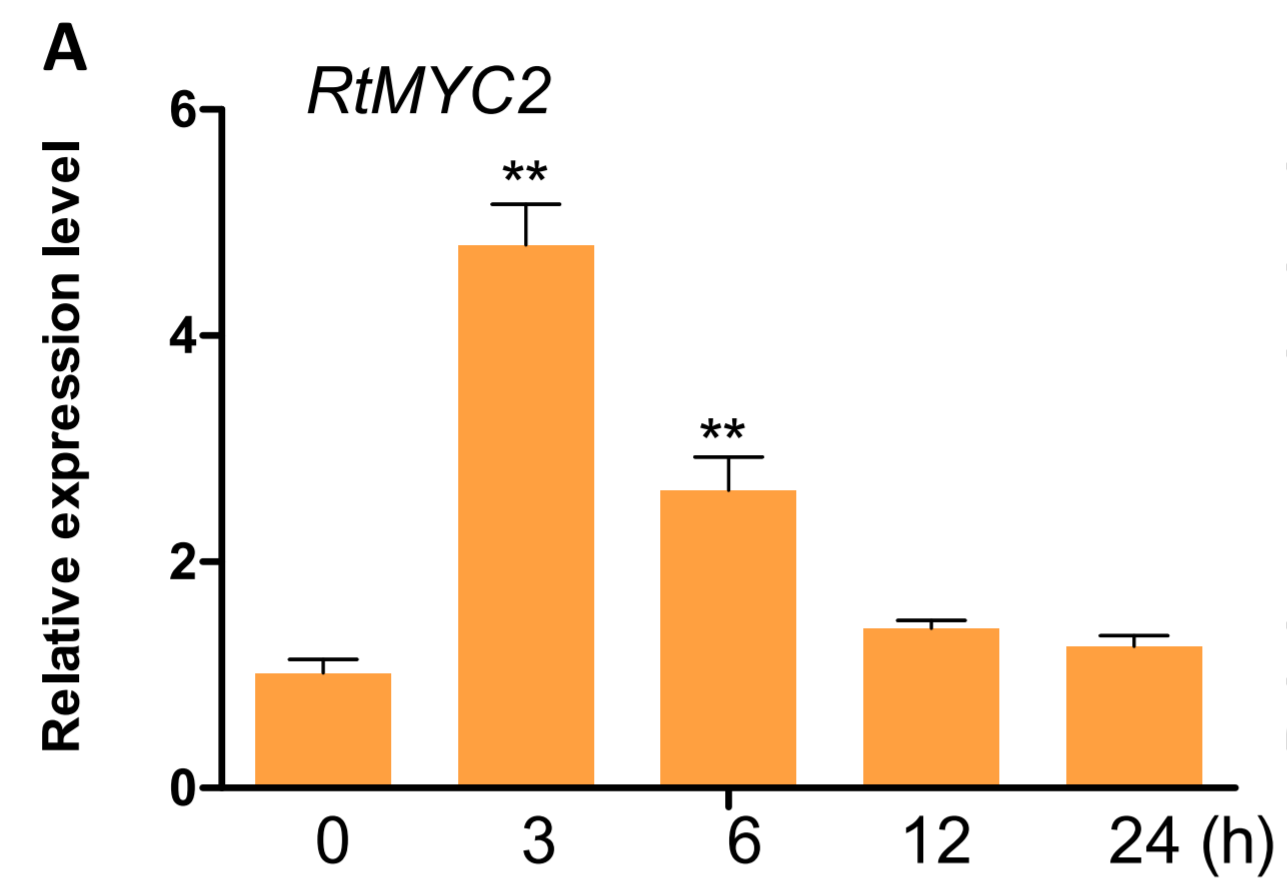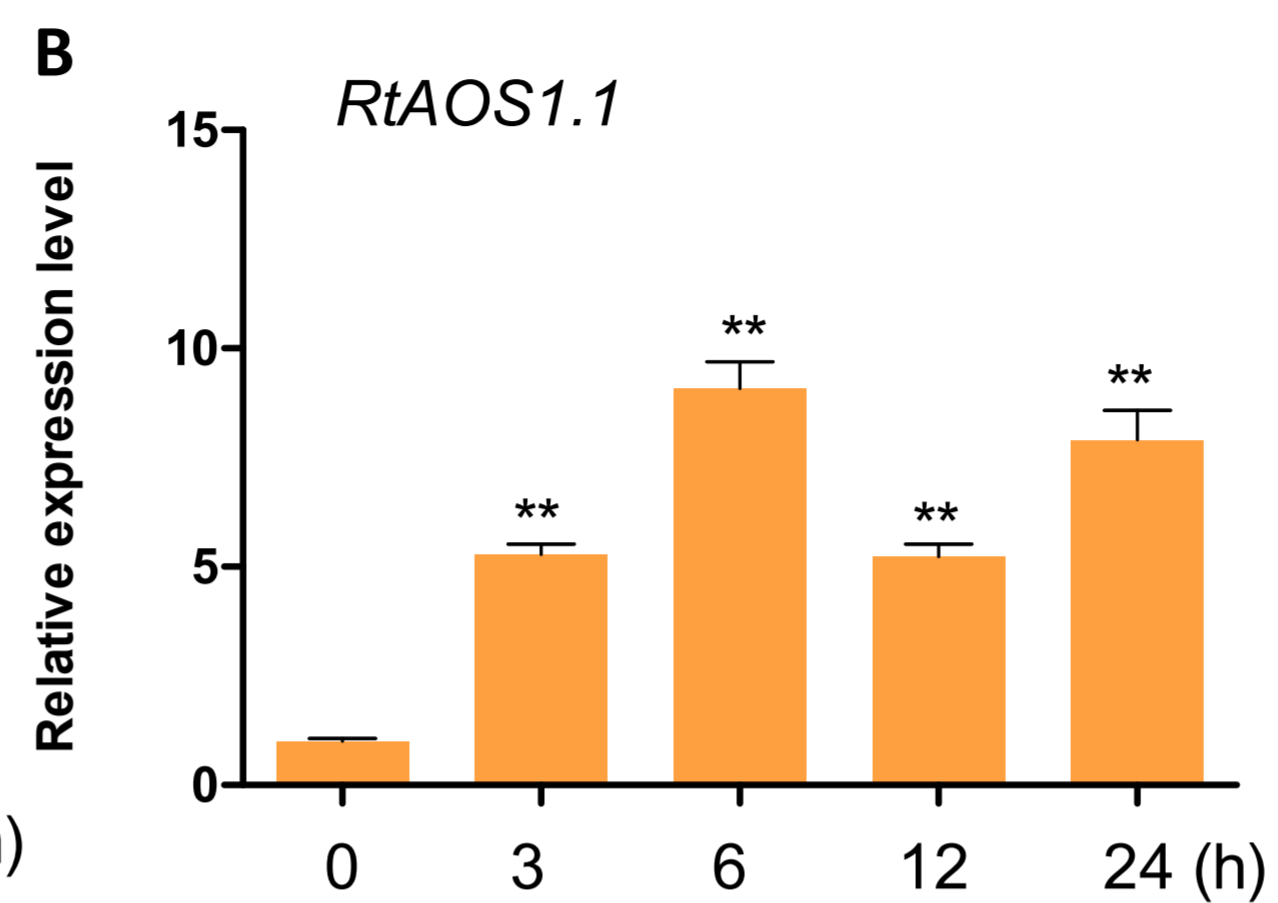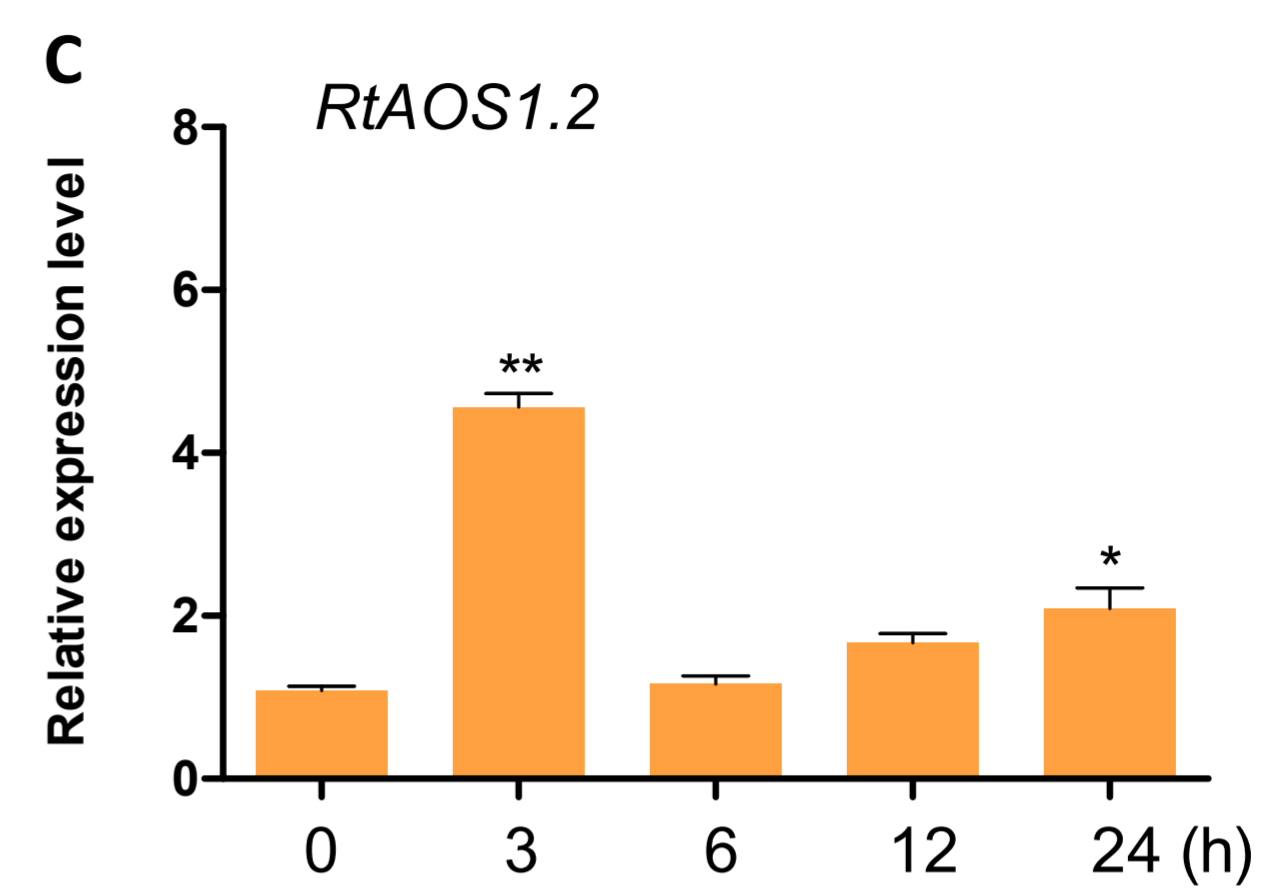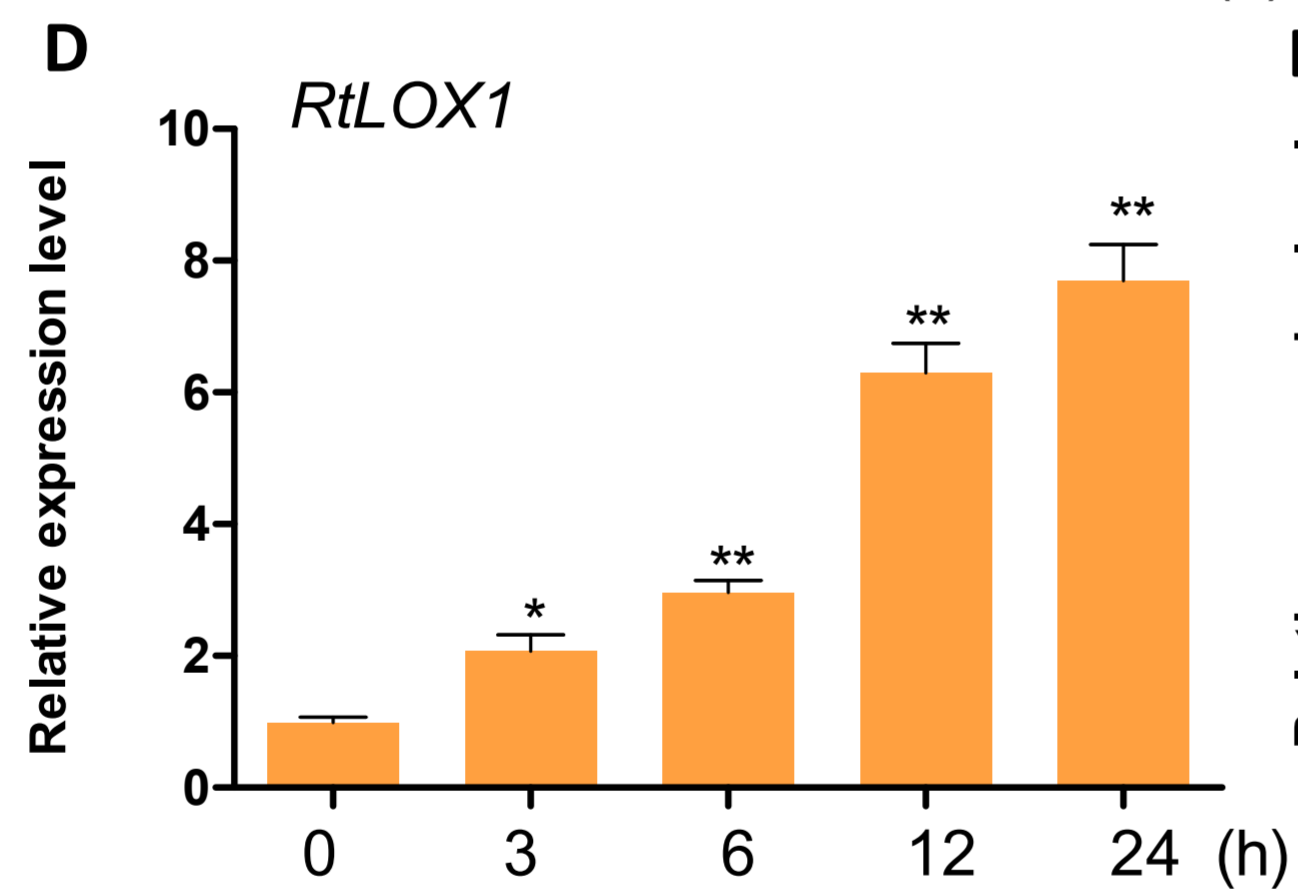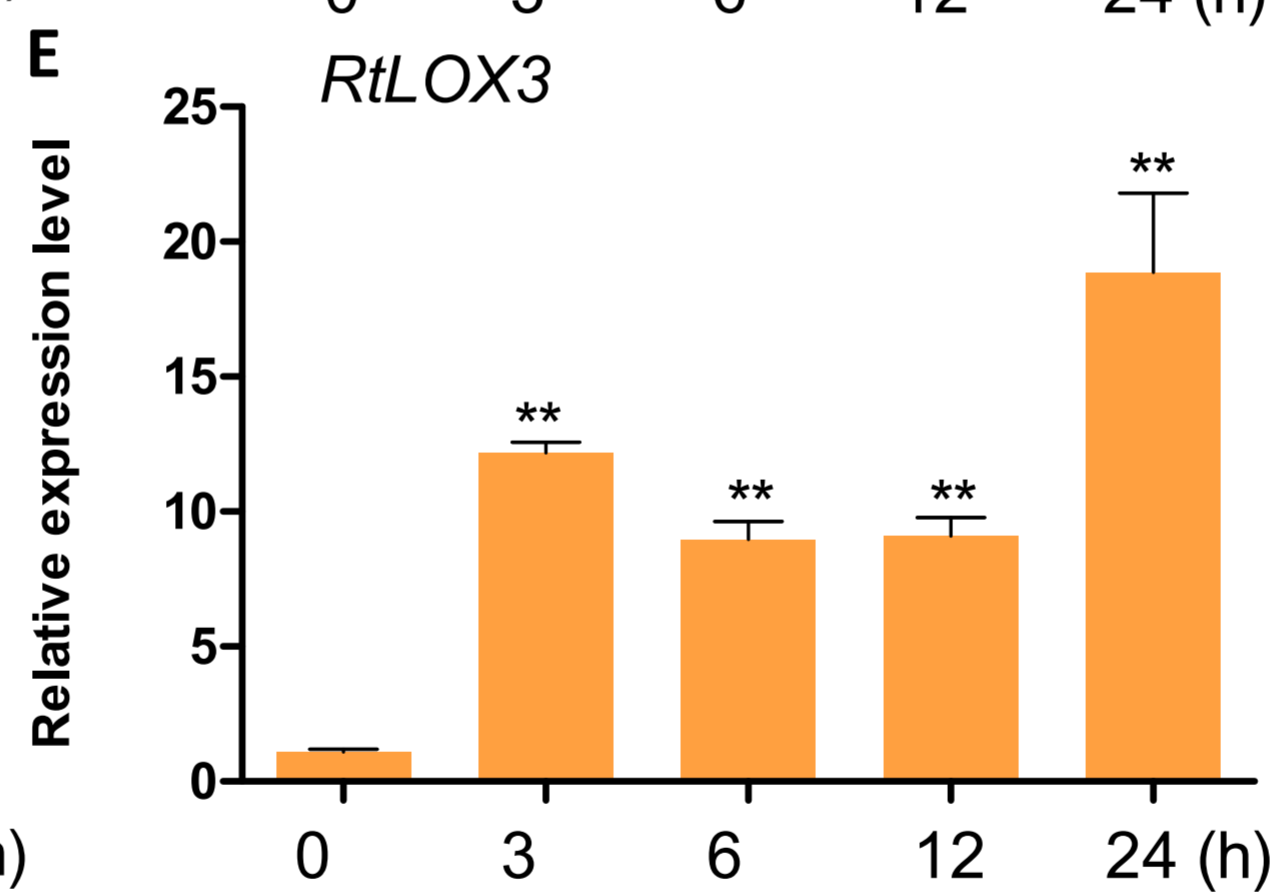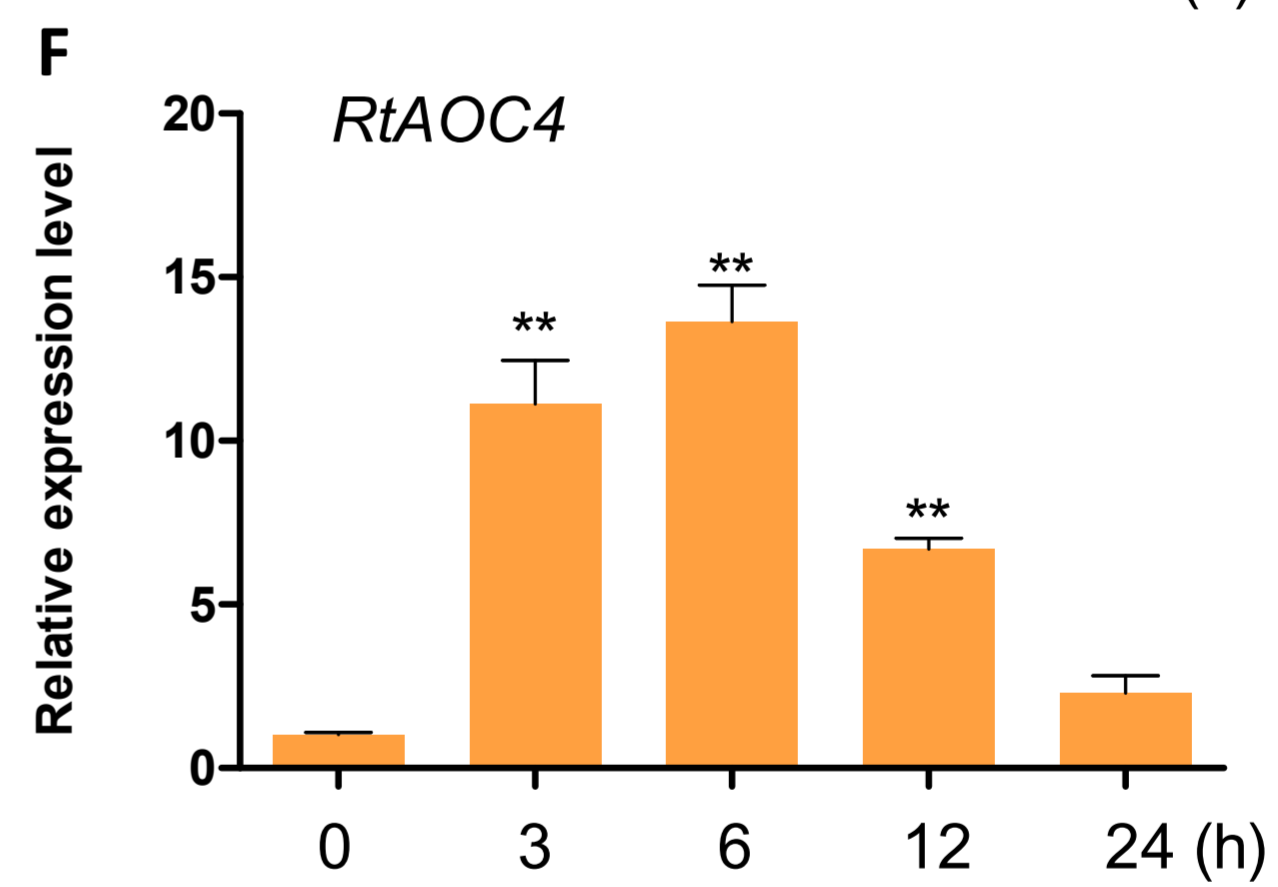

Supplement: Web_Material_uhae001 [file web_material_uhae001.zip › Fig S1.pdf]

A

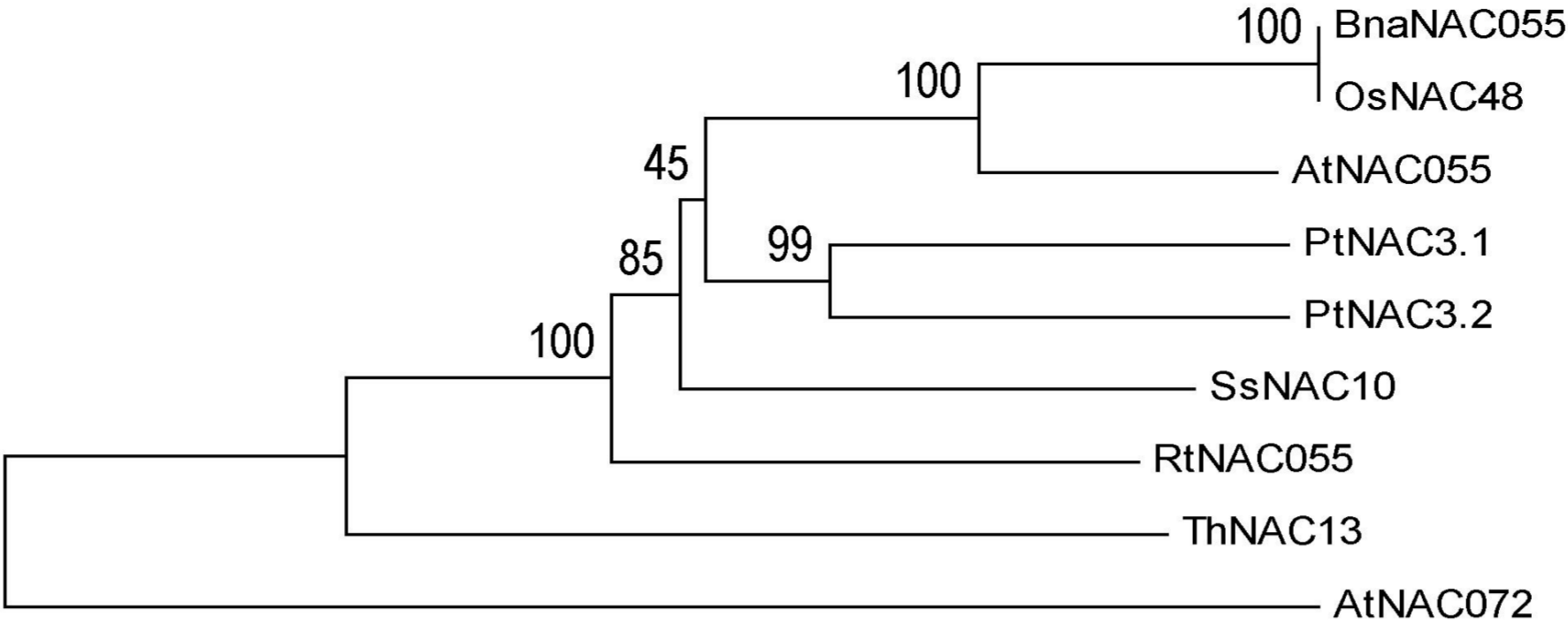

B

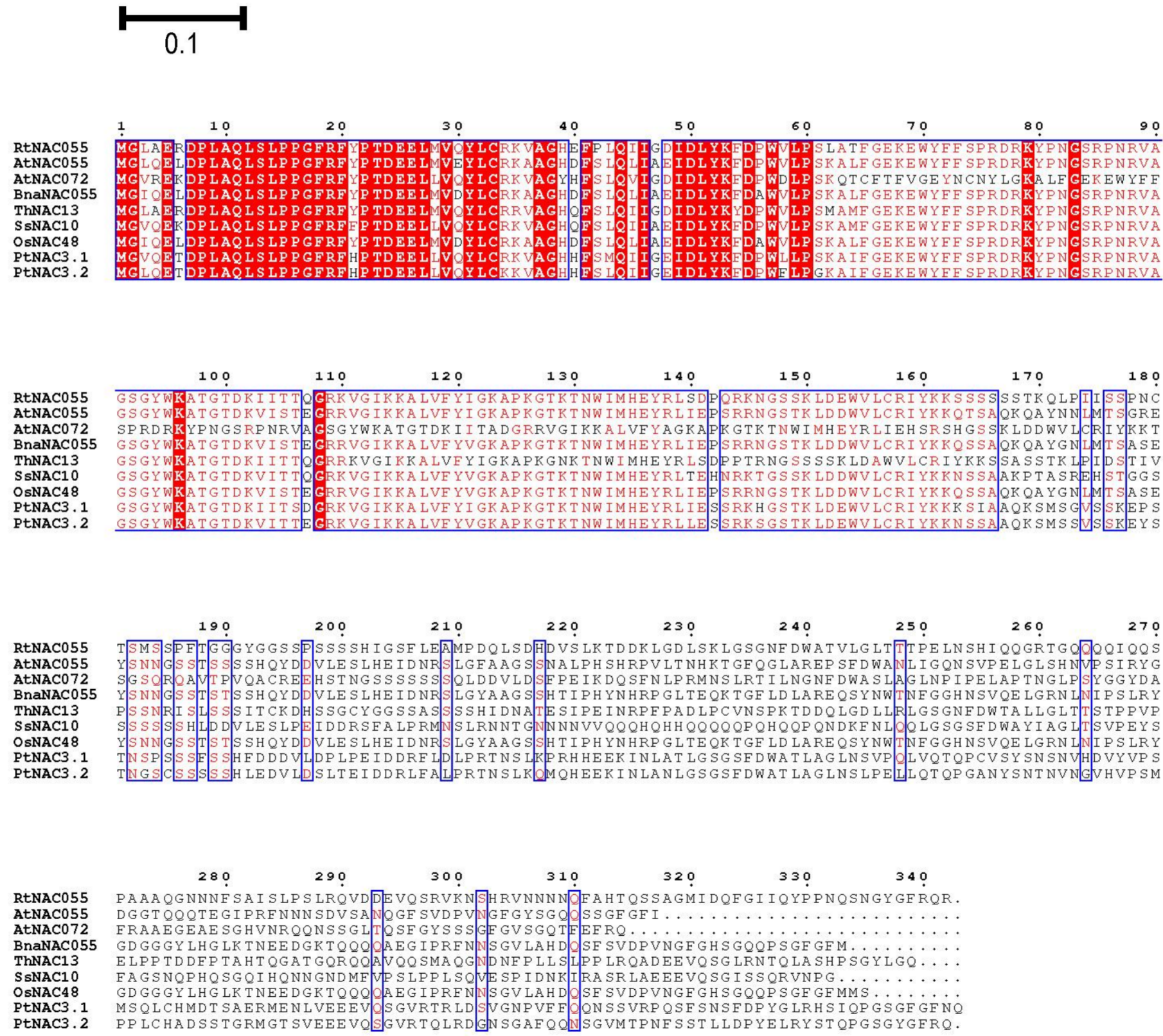

Supplement: Web_Material_uhae001 [file web_material_uhae001.zip › Fig S2.pdf]

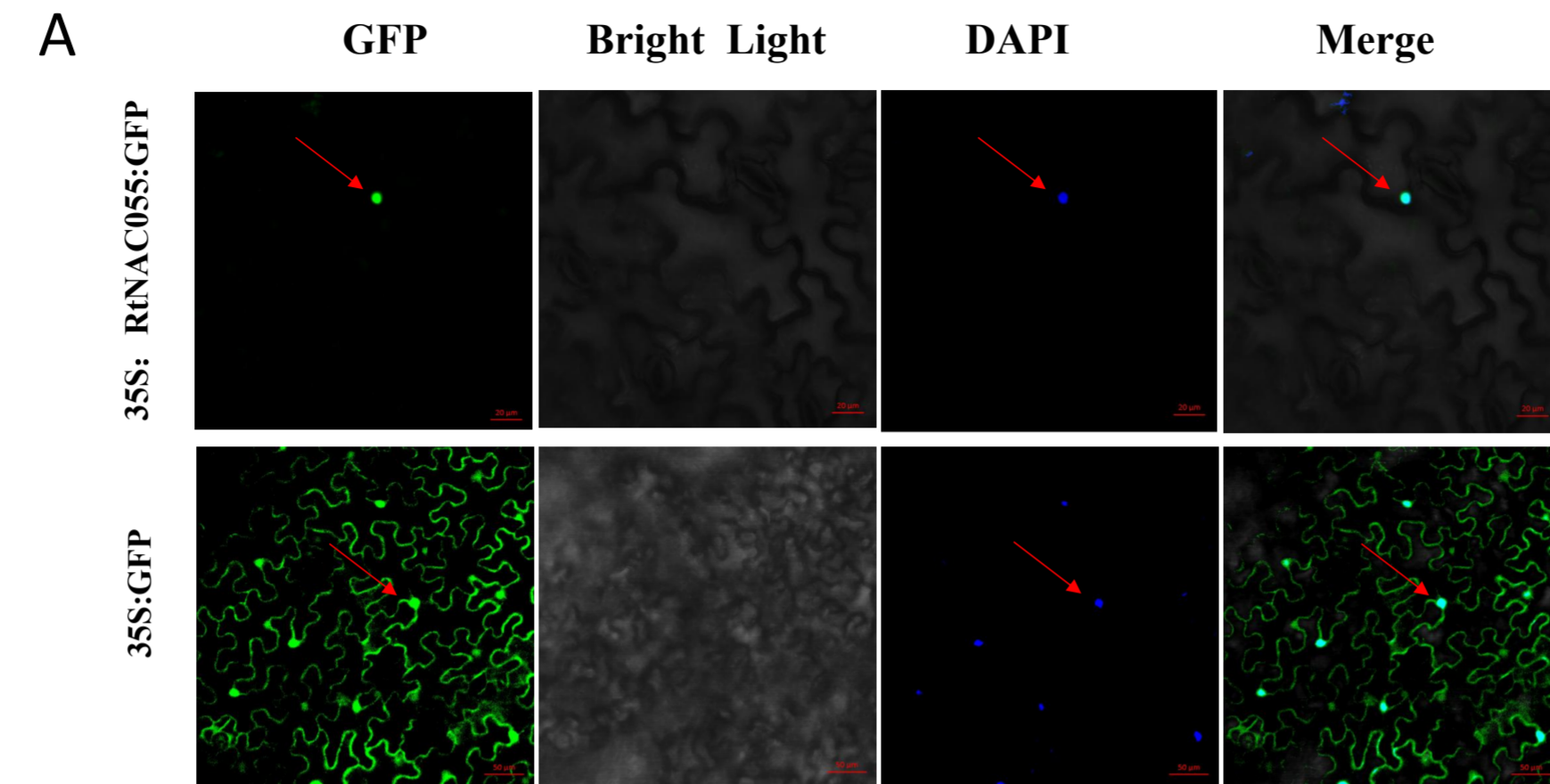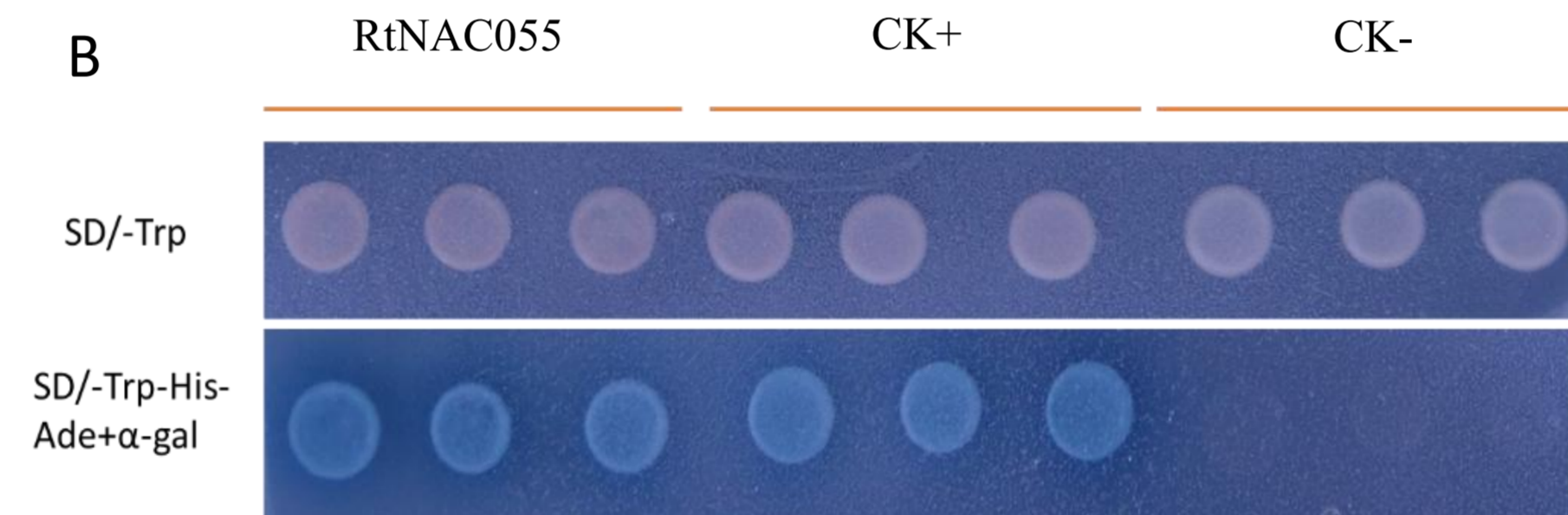

Supplement: Web_Material_uhae001 [file web_material_uhae001.zip › Fig S4.pdf]

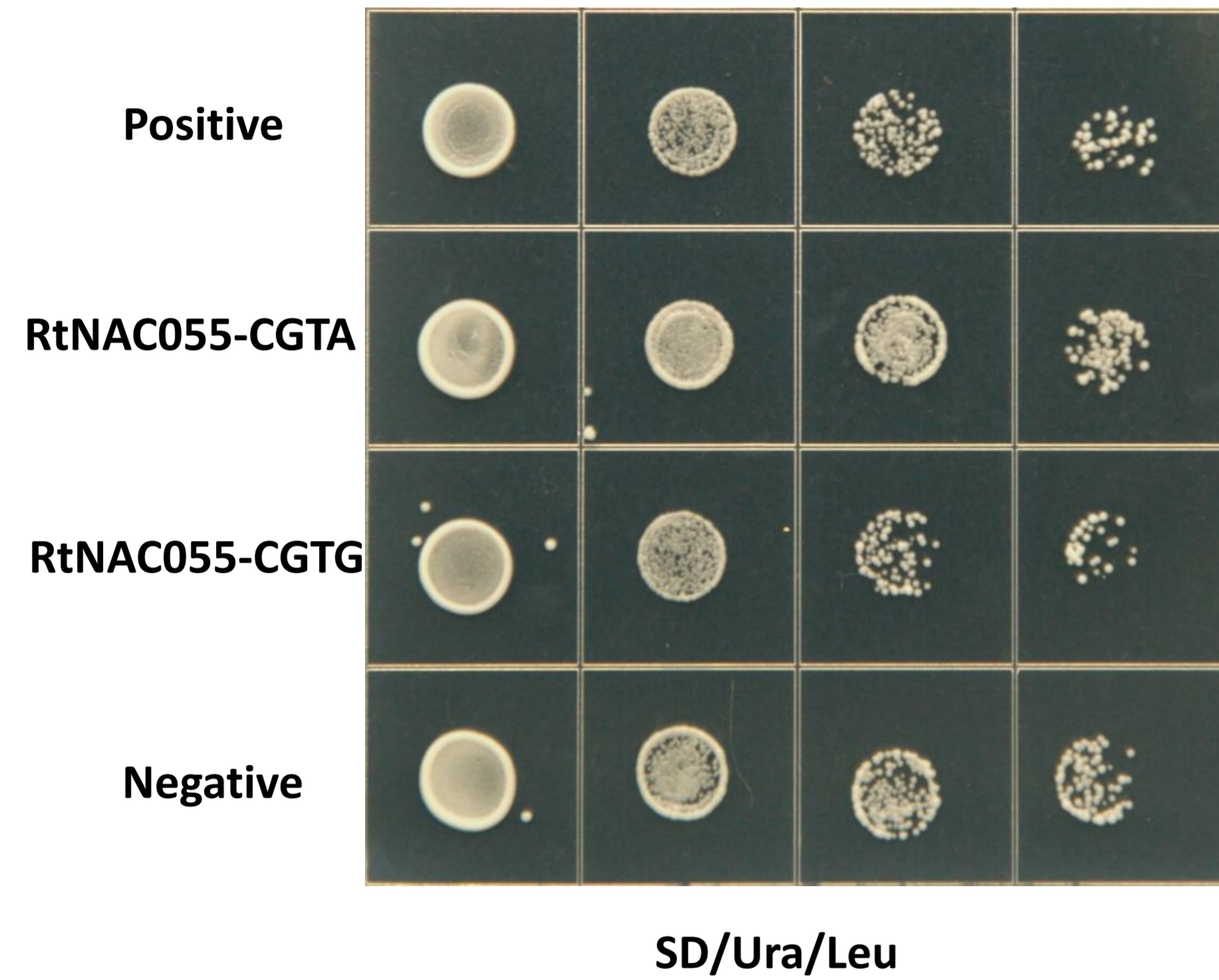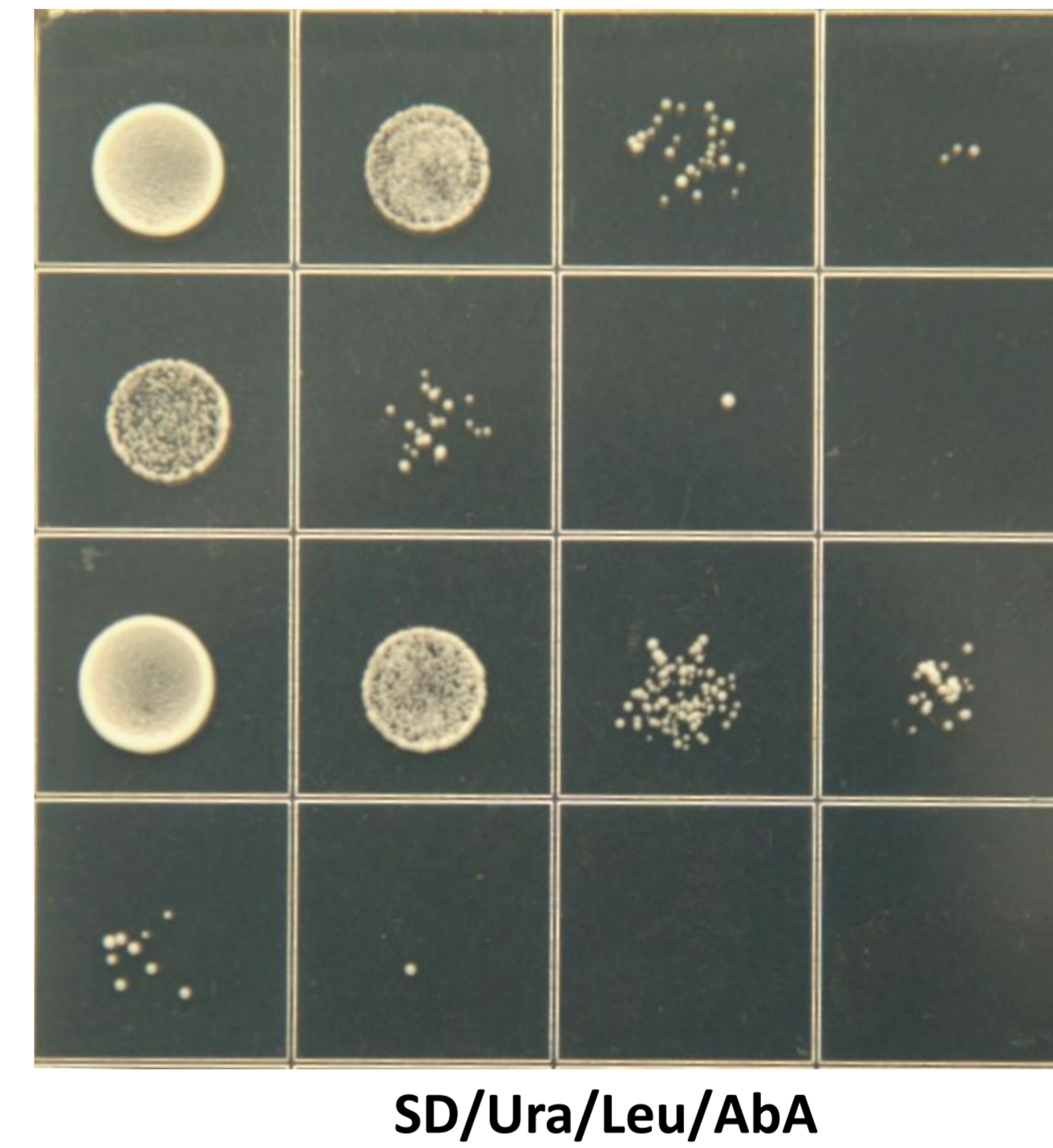

Supplement: Web_Material_uhae001 [file web_material_uhae001.zip › Fig S5.pdf]

WT

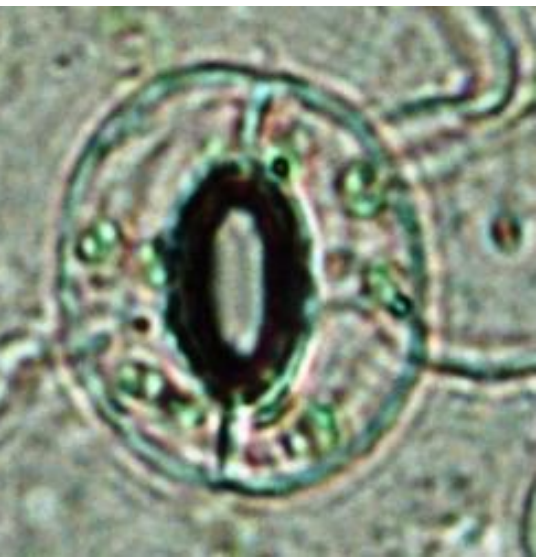

*atnac055*

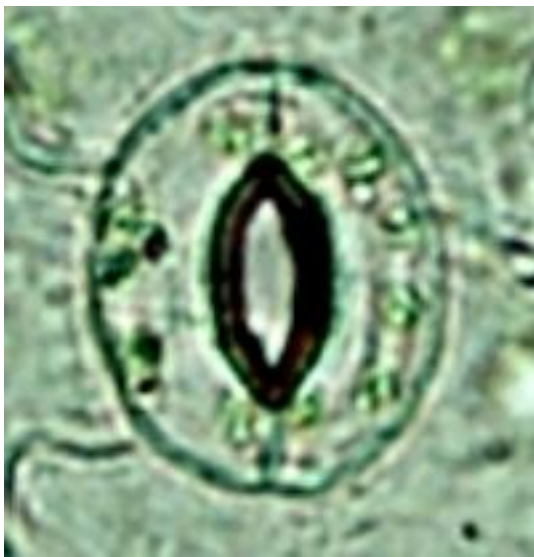

*atnac055/Rt*  
*NAC055#3*

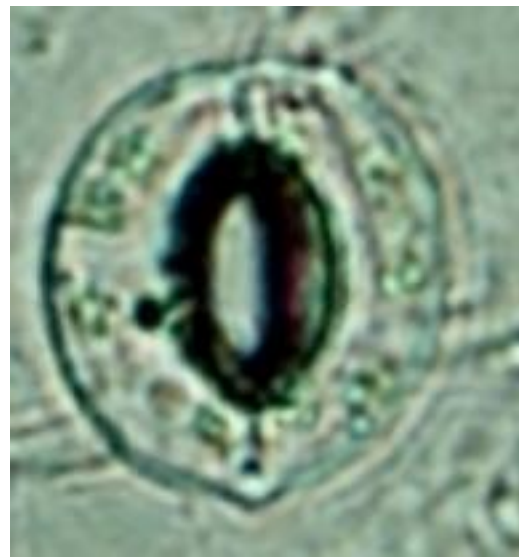

*atnac055/Rt*  
*NAC055#4*

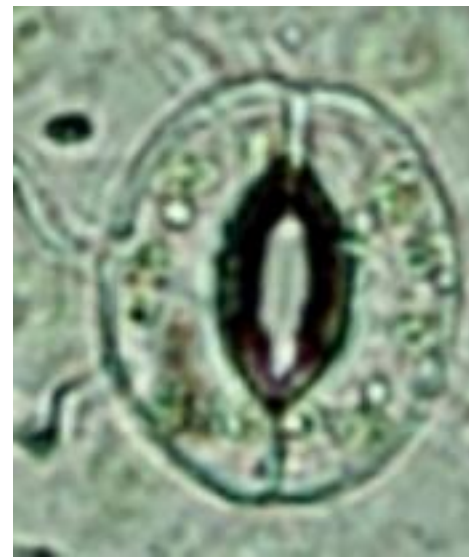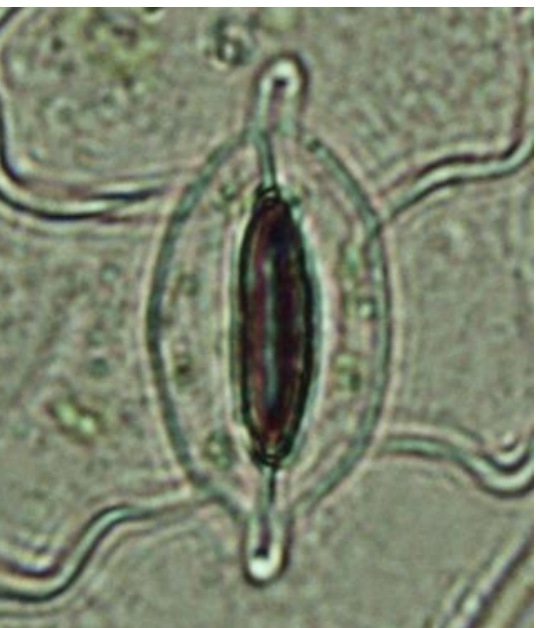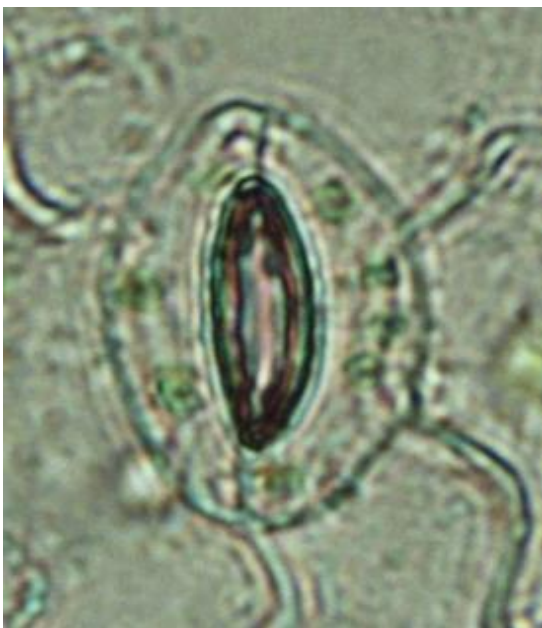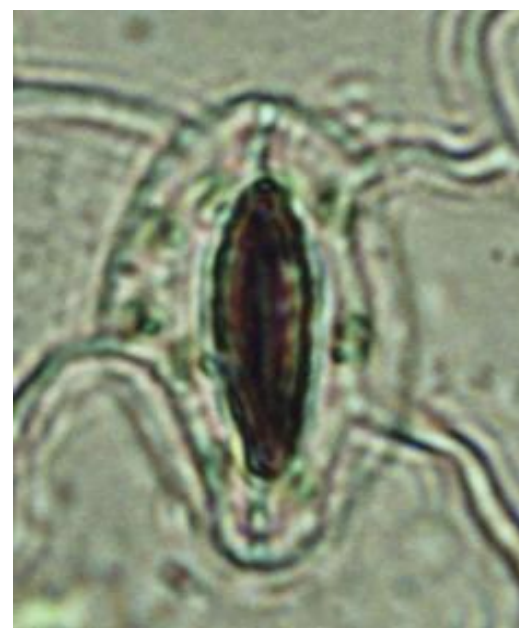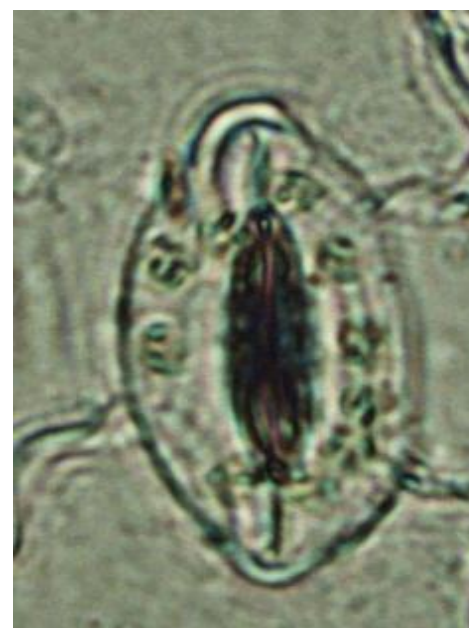

Supplement: Web_Material_uhae001 [file web_material_uhae001.zip › Fig S6.pdf]

**A*****RtDREB1.1***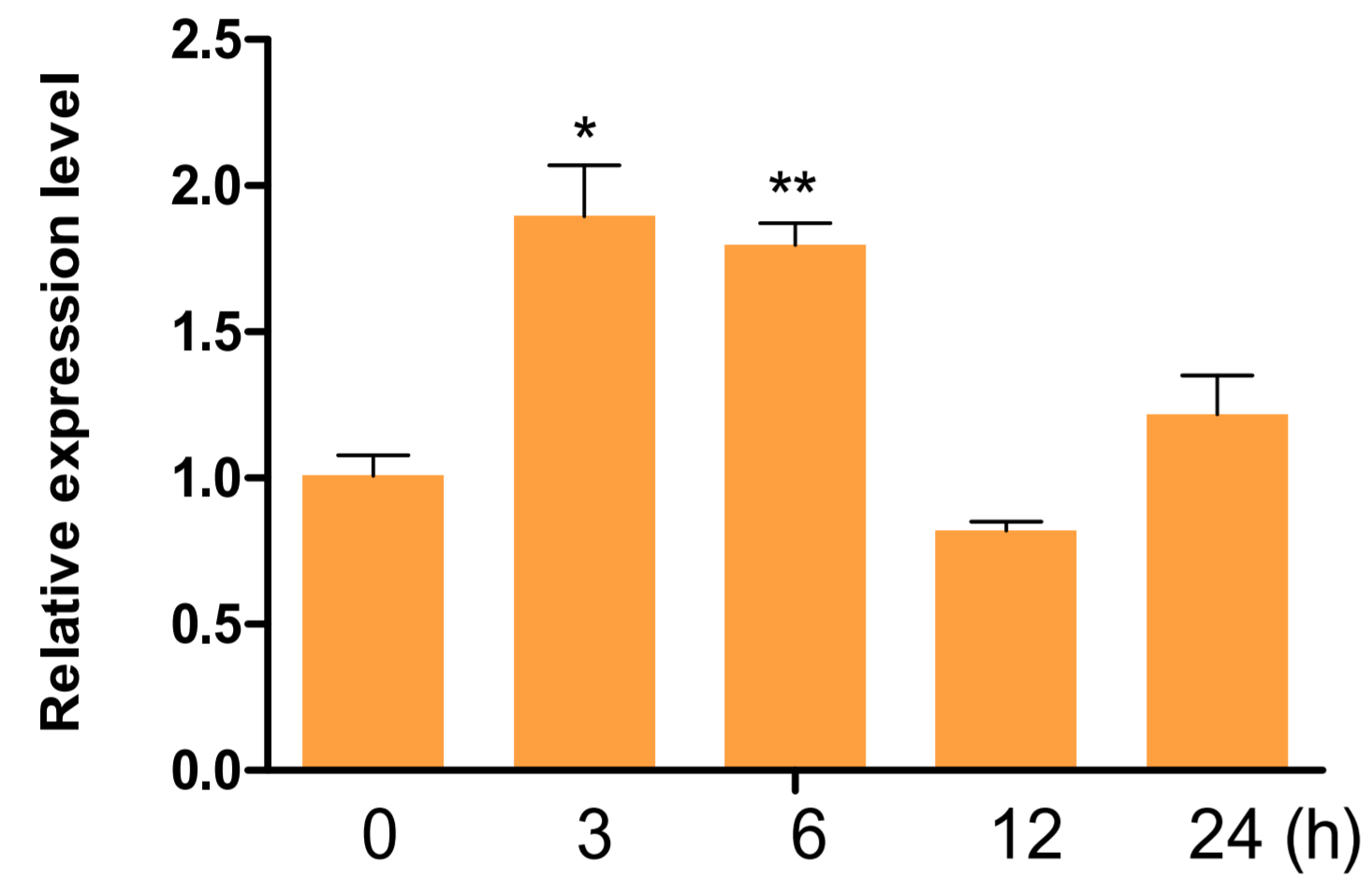**B*****RtRbohE***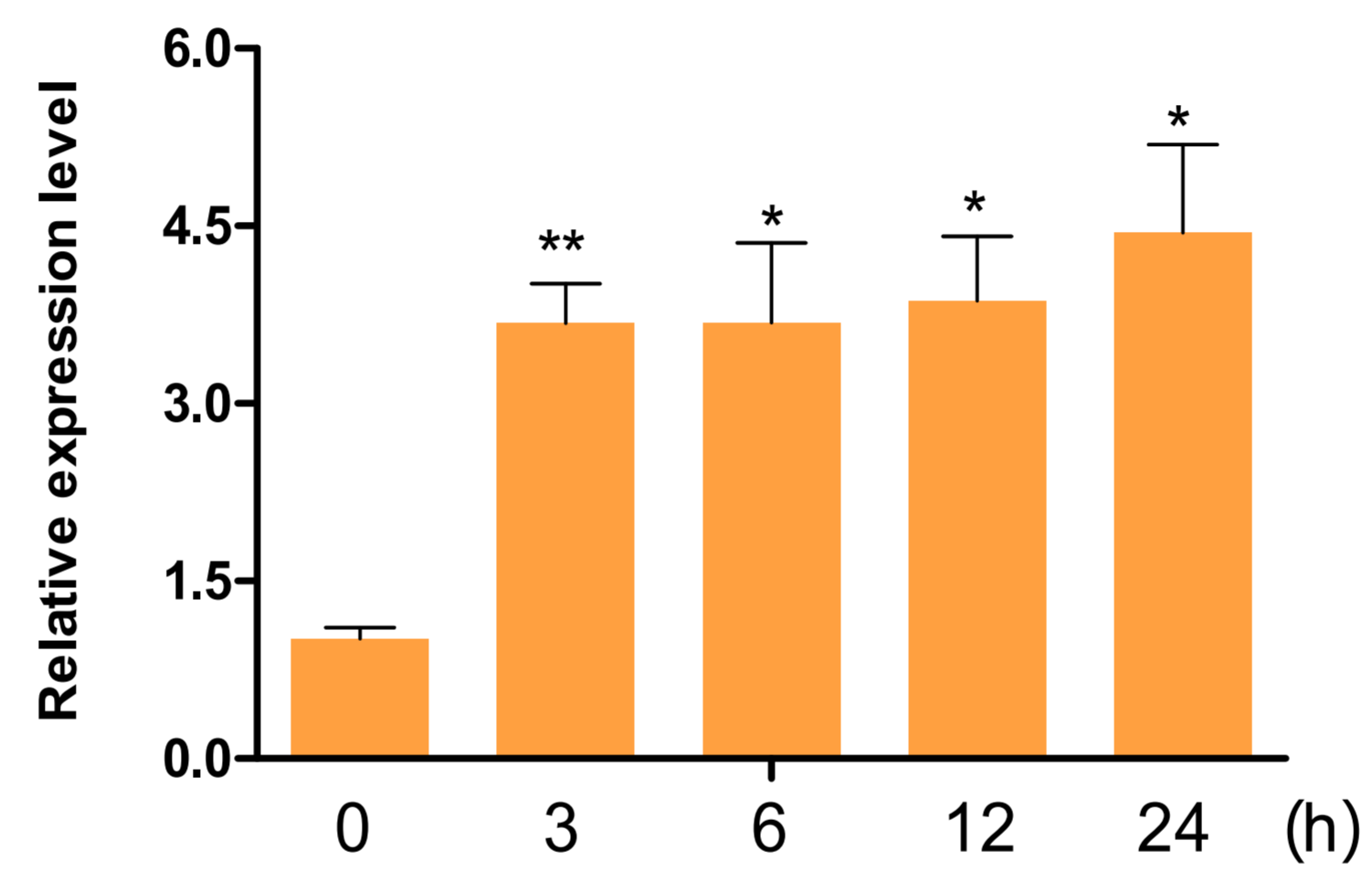**C*****RtP5CS1***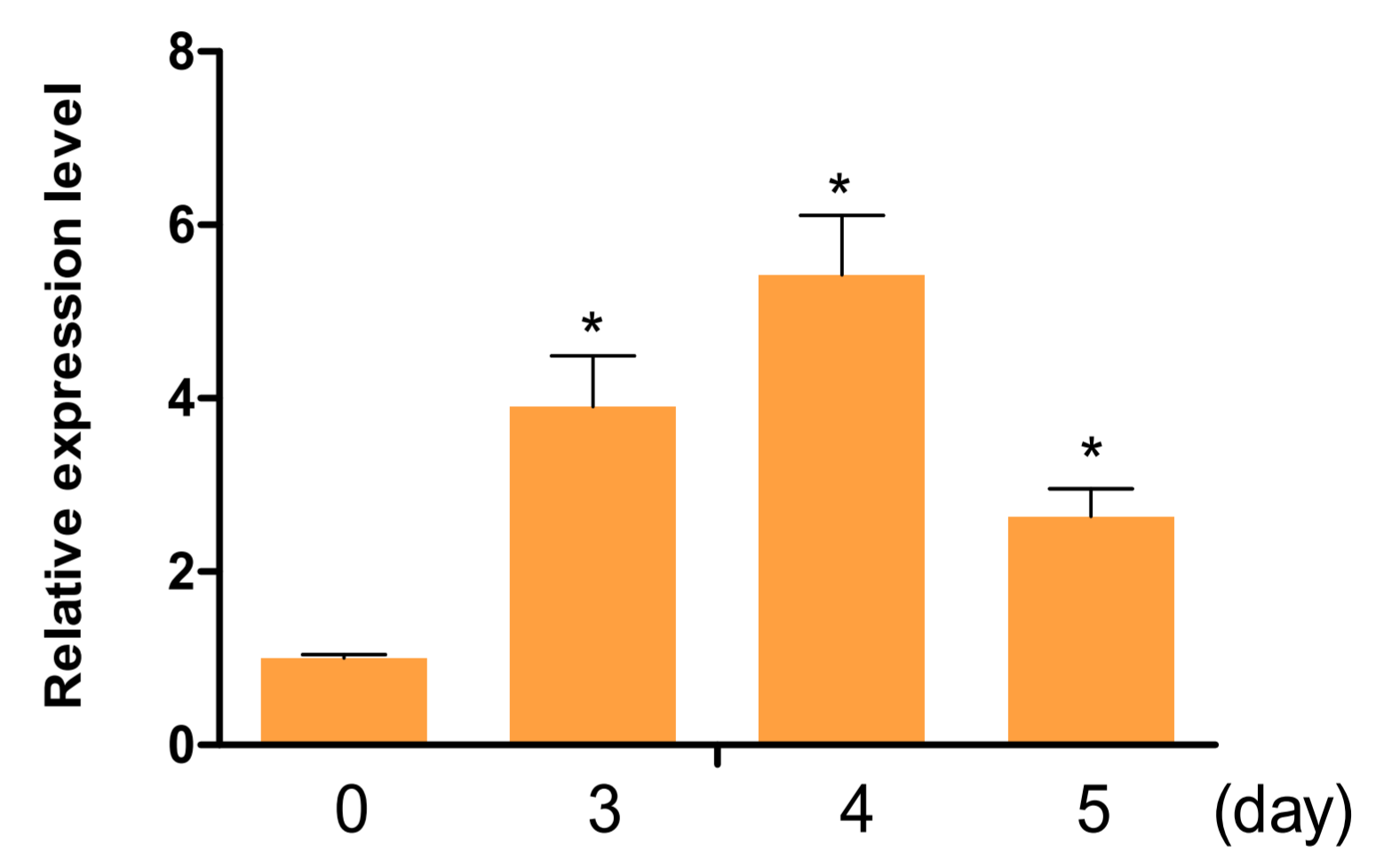

Supplement: Web_Material_uhae001 [file web_material_uhae001.zip › Fig S7.pdf]

**A**

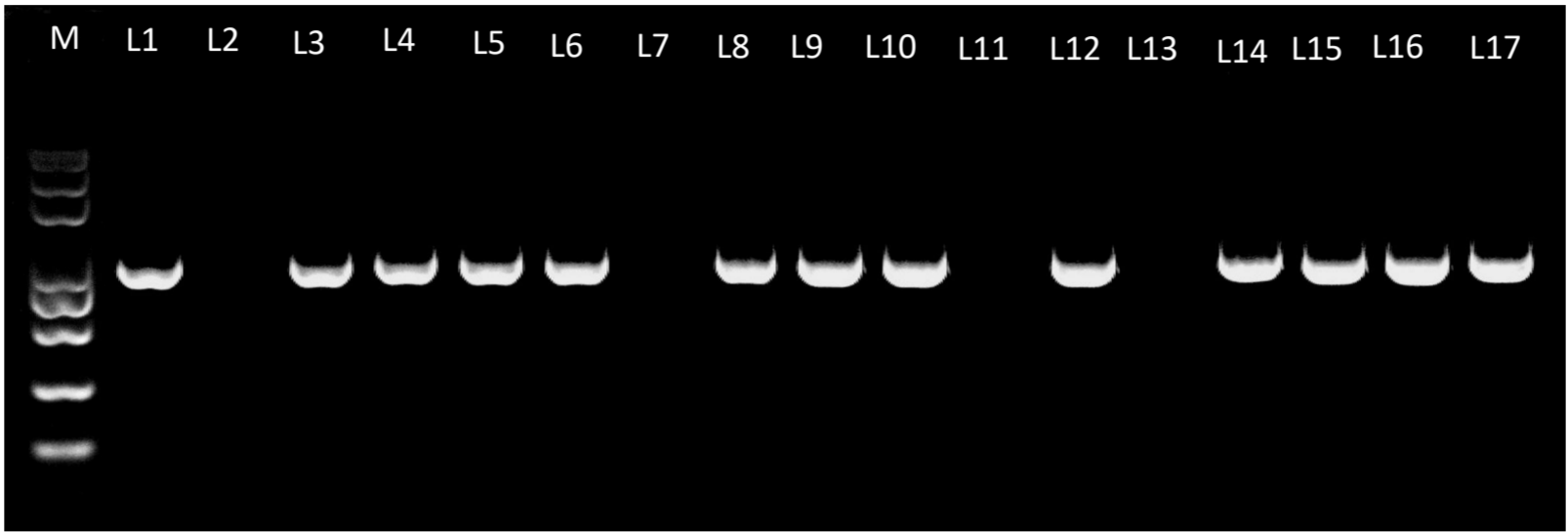

**B**

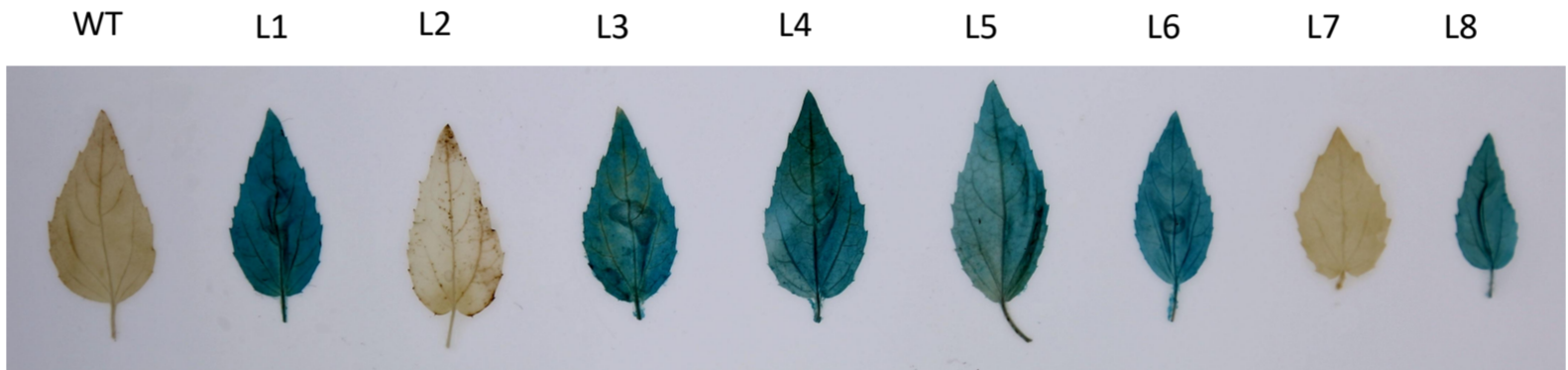

**C**

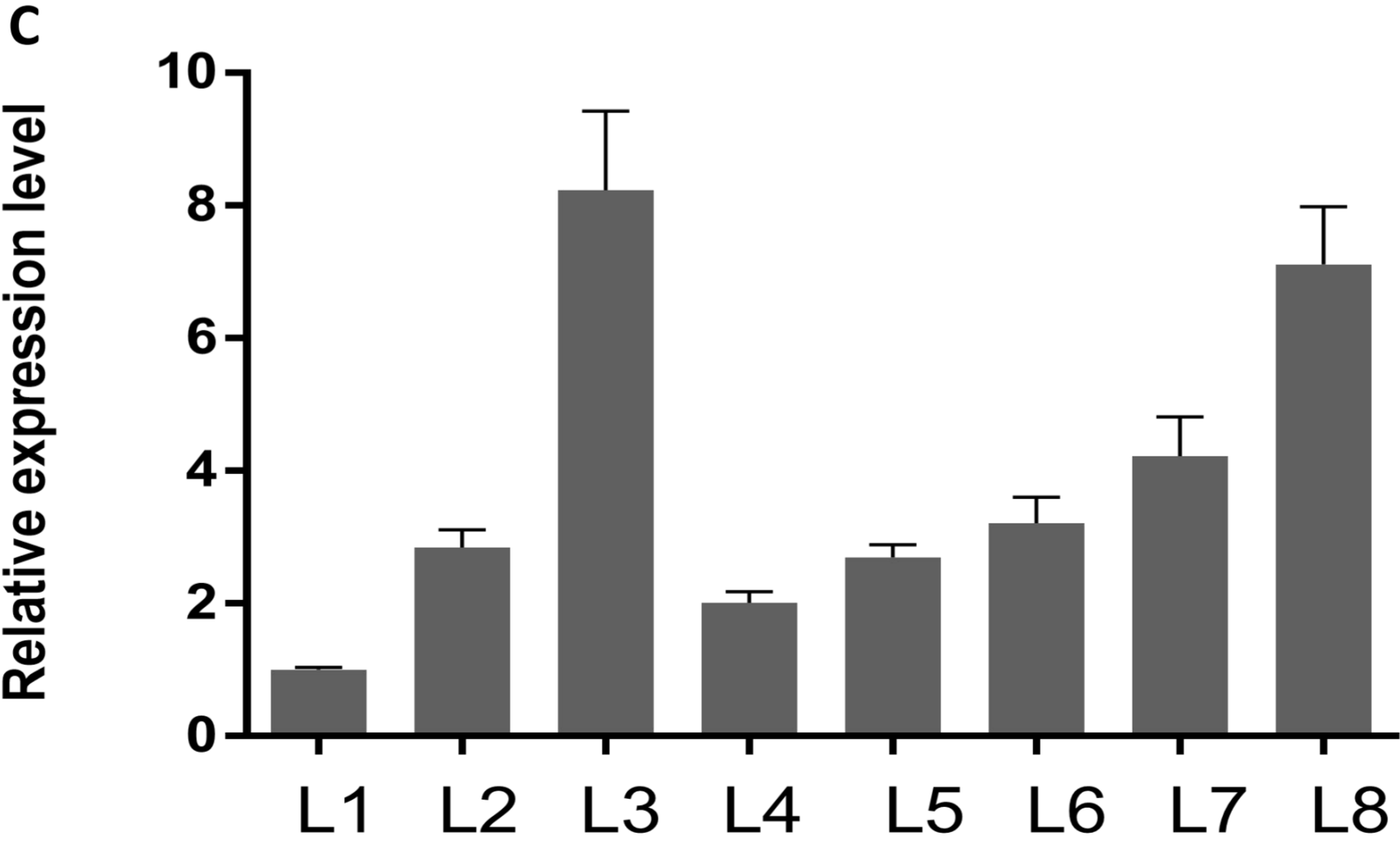

Supplement: Web_Material_uhae001 [file web_material_uhae001.zip › Fig S8.pdf]
